# Tailoring FPOX enzymes for enhanced stability and expanded substrate recognition

**DOI:** 10.1038/s41598-023-45428-1

**Published:** 2023-10-30

**Authors:** Hajar Estiri, Shapla Bhattacharya, Jhon Alexander Rodriguez Buitrago, Rossella Castagna, Linda Legzdiņa, Giorgia Casucci, Andrea Ricci, Emilio Parisini, Alfonso Gautieri

**Affiliations:** 1 Department of Biotechnology, Latvian Institute of Organic Synthesis, Aizkraukles 21, Riga, 1006 Latvia; 2 Faculty of Materials Science and Applied Chemistry, Riga Technical University, Paula Valdena 3, Riga, 1048 Latvia; 3 Dipartimento di Chimica, Materiali e Ingegneria Chimica “Giulio Natta”, Politecnico di Milano, Piazza L. da Vinci 32, 20133 Milano, Italy; 4 Biomolecular Engineering Lab, Dipartimento di Elettronica, Informazione e Bioingegneria, Politecnico di Milano, Piazza Leonardo da Vinci 32, 20133 Milano, Italy; 5 Department of Chemistry “G. Ciamician”, University of Bologna, Via Selmi 2, 40126 Bologna, Italy

**Keywords:** Enzymes, Protein design

## Abstract

Fructosyl peptide oxidases (FPOX) are deglycating enzymes that find application as key enzymatic components in diabetes monitoring devices. Indeed, their use with blood samples can provide a measurement of the concentration of glycated hemoglobin and glycated albumin, two well-known diabetes markers. However, the FPOX currently employed in enzymatic assays cannot directly detect whole glycated proteins, making it necessary to perform a preliminary proteolytic treatment of the target protein to generate small glycated peptides that can act as viable substrates for the enzyme. This is a costly and time consuming step. In this work, we used an in silico protein engineering approach to enhance the overall thermal stability of the enzyme and to improve its catalytic activity toward large substrates. The final design shows a marked improvement in thermal stability relative to the wild type enzyme, a distinct widening of its access tunnel and significant enzymatic activity towards a range of glycated substrates.

## Introduction

The current clinical standard for monitoring blood glucose levels is glycated hemoglobin (HbA1c). Indeed, thanks to its longevity (its half-life is up to 3 months), HbA1c is the marker of election for diabetes diagnosis. To this end, an effective protocol that includes the exploitation of fructosyl peptide oxidases (FPOX) for measuring HbA1c concentration in blood samples has been developed^1–3^. The first step of the protocol involves a proteolytic digestion of HbA1c to release the glycated N-terminal dipeptide, fructosyl-valine-histidine (fVH); the dipeptide is then hydrolyzed by a FPOX, a process that results in the production of hydrogen peroxide, which can be measured via a colorimetric assay by coupling horseradish peroxidase and an appropriate chromophore^4^. Likewise, FPOX-based methods can also be used to measure glycated albumin (GA), which is a short- and medium-term glycemic diabetes marker (half-life up to 3 weeks)^5^.

FPOXs are suitable enzymes for diabetes assays thanks to their reactivity on up to six amino acid-long fructosyl peptides^6^. FPOxs belong to the larger family of Fructosyl Amino Acid Oxidases (FAOX)^7–9^, which are enzymes of fungal or bacterial origin that cleave low molecular weight Amadori products, freeing the amine group and generating glucosone and hydrogen peroxide^10–12^.

Other than diabetes detection and surveillance, FPOXs are also considered to be promising therapeutic tools for protein deglycation in biological tissues^13^. Protein glycation is a spontaneous and irreversible non-enzymatic reaction whereby a sugar binds to a protein and generates a covalent adduct named Amadori product^14^. Protein glycation triggers many diabetes-related clinical outcomes, such as for instance arterial stiffening^15^, nephropathy^16^, retinopathy^17^ and neuropathy^18^. These complications have indeed been shown to be largely driven by the chemical modification of functional proteins^19,20^.

The inability of wild type FPOXs to act on intact proteins is the most critical issue limiting both their diabetes detection efficiency and their protein deglycation potential^21,22^. This is due to their buried active site and narrow access tunnel, as determined in multiple crystal structures^6,12,23^. To the best of our knowledge, there is currently only one report describing an enzyme that shows direct activity on HbA1c without requiring any preliminary proteolytic step. This enzyme has been obtained by introducing a single point mutation (R61G) in a thermostable version of *Aspergillus nidulans'* FPOX (AnFPOX)^24^. Nonetheless, the activity and the stability profile of FPOX enzymes need to be further improved when considering their application in biosensors. Indeed, improving the ability of FPOXs to act on full-length glycated proteins may provide significant advantages in both the development of novel diabetes monitoring tools and in the design of innovative therapies aimed at reducing and/or preventing protein aging (i.e., the accumulation over time of advanced glycation end-products). These improvements may come from engineering an enzyme with a wider access tunnel to the catalytic site; in fact, access tunnel engineering can improve several characteristics of the enzyme, such as activity, specificity, promiscuity, enantioselectivity and stability^25,26^.

Our group has previously reported on a successful engineering campaign aimed at widening the access tunnel to the catalytic site of *Phaeosphaeria nodorum* FPOX (PnFPOX)^27^. In that account, using a rational design approach involving a five-amino acid deletion at the entrance of the active site and a number of further mutations, we described a stable PnFPOX mutant (named L3-35A) that, relative to the wild type enzyme, features a significantly wider and shorter access tunnel. However, the backbone redesign also introduced structural instability and lowered the activity of the engineered enzyme. For this reason, in this work we aimed at improving the L3-35A design and restore the stability and activity of the enzyme, thus providing a FPOX that presents high activity and stability together with a wider access tunnel with respect to the parental enzyme PnFPOX.

In the present study, we set out to enhance the thermal stability of the L3-35A mutant by implementing a methodological approach whereby different thermal stabilization strategies are tested in parallel and their efficiency compared. These various strategies involved mutations to either improve the RMSF of the protein (generating mutants hereafter referred to as D-series mutants), increase the number of salt bridges (C-series mutants) or form disulfide bonds (X-series mutants). Whereas, of all methods, engineered disulfide bond formation usually provides the enzyme with the highest T_m_, the other two methods may in fact be useful when engineering a protein that already contains one or more cysteines. In such cases, the introduction of further cysteine residues may interfere with natural disulfide bond patterns and result in high levels of insoluble protein formation. In essence, we used our PnFPOX mutant L3-35 as a platform to test our design approach on different thermal stabilization strategies, and to provide a systematic comparison of their efficiency relative to the same parent enzyme. Our design process involved the use of extensive molecular dynamics (MD) simulations and targeted optimization using Rosetta-based design^28^, followed by experimental validation of a small number of selected high-scoring mutants. In our validation procedure, we assessed the activity of the selected mutants against several substrates, including small glycated amino acids such as fructosyl-Val (fV) and fructosyl-Lys (fK), the dipeptide fructosyl-Val-His (fVH) and the hexapeptide fructosyl-Val-His-Leu-Thr-Pro-Glu (F6P). Overall, the data shown herein provide evidence that our rational design approach allows the rapid identification of mutants featuring the desired phenotype.

## Results and discussion

### Stability optimization of the L3-35A PnFPOX mutant

**Figure 1:**
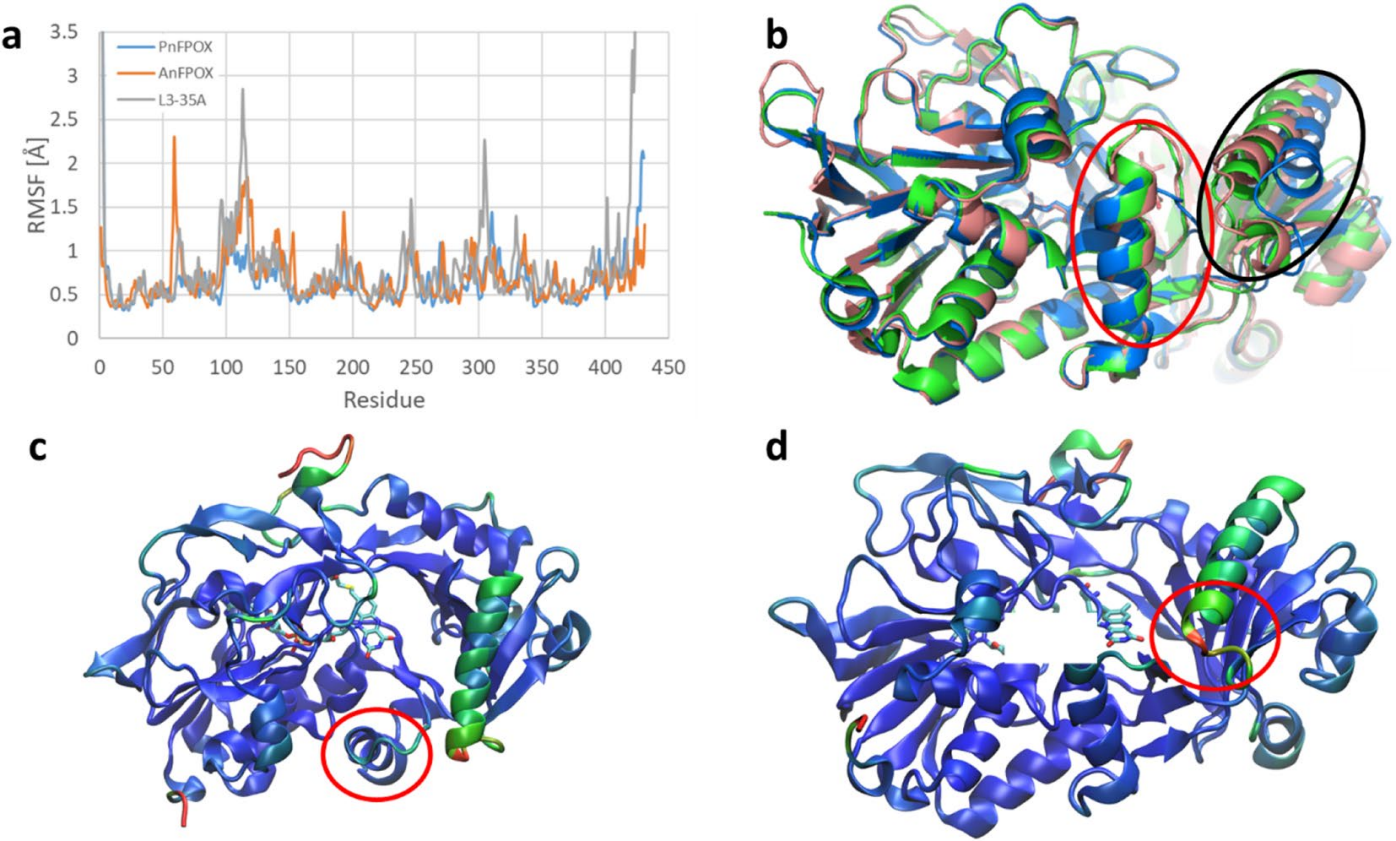
Stability analysis from MD simulations. The RMSF profile (**a**) shows that in the L3-35A, the helix 96–111 and the surrounding structures present a high degree of instability compared to the other enzymes. In the structural comparison (**b**, PnFPOX:green, AnFPOX:pink and L3-35A:blue), this helix (circled black) shows also a different fold compared to the other two enzymes. The instability and different structural arrangement could be due to design operated on L3-35A, which removed part of the helix blocking the tunnel (**c**, circled red). These removed residues were facing the helix 96–111 in the parental enzymes and were making stabilizing interactions, which could explain why helix 96–111 is unstable in L3-35A, possibly leading to its lower activity (**d**, color coded by RMSF, where blue represents lower RMSF and green–red the regions with higher RMSF). Comparison of RMSF and structure of PnFPOX (green), AnFPOX (pink) and L3-35A (blue).

Starting from the crystal structure of the L3-35A PnFPOX mutant (PDB code 6Y4J), we performed MD simulations at variable temperatures, followed by root mean square fluctuation (RMSF) analysis. Our data suggests that, with the exception of the N- and the C-termini, the most unstable regions of the enzyme are helix 96-111 and the surrounding structures (Fig. 1). The relative instability of this helix is likely due to the redesign of residues 58–65 of wild type PnFPOX (red circle in Fig. 1c)^27^. Indeed, while these changes resulted in the widening of the access tunnel to the active site, they also caused the removal of the amino acids facing helix 96-111, thus abrogating previously existing stabilizing interactions. Further regions that present a lower average stability are loops 240–247, 301–307, 401–410.

The use of the Rosetta “Supercharge” method, which aimed at increasing the charge on the surface of the enzyme, led to the design of 1000 enzyme variants, from which those 20 (named C01–C20) that featured the best Rosetta score were selected for further analysis. Similarly, single point mutations screening led to 8095 designs, from which the best 20 were retained (S01–S20). Double point mutations screening generated 271,000 designs (leading to the selection D01–D20), PROSS design produced 9 variants (P01–P09), and disulfide bond design identified 62 enzyme variants (from which we selected the best 8, named X01-X08).

The 77 candidate enzyme variants originating from our different screening methods were then subject to MD simulations and ranked based on their stability at increasing temperatures, as assessed by RMSF analysis. Our MD-based ranking led to the selection of six candidate mutants (C16, D02, X01, X02A, X04, and X07) for experimental production and characterization.

For measuring the thermal stability of the different mutants, we used both thermal shift assay (differential scanning fluorimetry or DSF) and circular dichroism (CD). Both of these methods are sensitive to the thermal stability of the enzymes and can provide information on the structural changes that occur as a function of temperature. As expected, the two methodologies provide very similar results in terms of trends and absolute values and demonstrate that the rational design procedure that we adopted was effective in enhancing the thermal stability (T_m_) of our reference FPOX enzyme, L3-35A (see Table 1). The D02 variant, which relative to L3-35A features two additional mutations (V110R and D115G), shows an increase in T_m_ of ≈ 1.5 °C with respect to the parent enzyme. On the other hand, the introduction of extensive surface charges and salt bridges (mutant C16) resulted in a further increase in T_m_ of ≈ 1.5 °C (T_m_ = 55.2 °C). Finally, the incorporation of a single disulfide bond in different positions on the L3-35A scaffold led to a significant improvement in thermal stability of all the tested mutants of this series: X01 (54.1 °C), X02 (60.0 °C), X04 (55.2 °C) and X07 (55.3 °C).

**Table 1:** Melting temperature of the parent enzyme (L3-35A) and its mutants as determined by thermal shift assay (^a^) and circular dichroism (^b^).

Enzyme	T_m_ (°C)^a^	T_m_ (°C)^b^
PnFPOX (WT)	53.2 ± 0.2	53.5 ± 0.1
L3-35A	52.3 ± 0.2	52.9 ± 0.1
D02	53.1 ± 0.5	54.8 ± 0.1
C16	55.2 ± 0.1	55.0 ± 0.1
X01	54.1 ± 0.1	54.2 ± 0.1
X02A	60.0 ± 0.4	60.1 ± 0.1
X04	55.2 ± 0.3	55.0 ± 0.1
X07	55.3 ± 0.4	55.4 ± 0.1
X02B	60.2 ± 0.7	60.6 ± 0.1
X02C	64.0 ± 0.2	63.3 ± 0.1

The melting temperature of the wild type enzyme (PnFPOX) is also provided as reference.

To assess the activity of these various enzyme variants, we employed a colorimetric enzymatic assay that allowed us to quantify the release of glucosone probed at 322 nm. We tested the activity of the enzymes on fructosyl-lysine (fK), fructosyl-valine (fV), fructosyl-valine-histidine (fVH) and fructosyl-Val-His-Leu-Thr-Pro-Glu (F6P) (see Table 2). We observed that the activity of the enzyme variants towards fK and fV remained similar to that of the parent enzyme, with a slight increase of activity in the case of D02 and a slight decrease in all the other cases. Overall, we observed that the activity of these mutant enzymes is several folds lower than that of PnFPOX, the naturally occurring enzyme. Also, similarly to the parent enzyme, none of these mutants shows any activity towards the dipeptide or the hexapeptide.

**Table 2:** Comparison of the enzymatic specific activity (U/mg) on different substrates.

Enzyme	Specific activity (U/mg)			
	fK	fV	fVH	F6P
PnFPOX	30.18 ± 0.67	29.67 ± 2.56	32.60 ± 1.18	0.78 ± 0.09
AnFPOX-47	–	–	–	0.082 ± 0.002^a^
L3-35A	0.21 ± 0.02	0.16 ± 0.04	–	–
D02	0.30 ± 0.03	0.32 ± 0.01	–	–
C16	ND	ND	–	–
X01	ND	ND	–	–
X02A	0.15 ± 0.01	0.11 ± 0.01	–	–
X04	ND	0.13 ± 0.01	–	–
X07	–	0.08 ± 0.01	–	–
X02B	2.24 ± 0.07	32.50 ± 0.44	0.87 ± 0.03	0.18 ± 0.06
X02C	1.06 ± 0.01	17.95 ± 2.46	1.62 ± 0.02	0.43 ± 0.06

Tests are performed in triplicates.

*ND* not detected.

^a^Data from reference^24^.

Based on our thermostability results, we selected the X02A variant for its high T_m_, high expression yield, and detectable activity toward glycated amino acids and we set out to increase its activity toward larger substrates (i.e., fVH and F6P). The activity toward these substrates was present in the wild type enzyme PnFPOX but was lost in the L3-35A design. The L3-35A design involved the engineering of the entry tunnel of the wild type PnFPOX enzyme via the shortening of a loop and a helix^27^. In this process, while the amino acids that are directly involved in the catalytic function were not modified, some tunnel-lining residues were replaced. This could explain the reduced activity of both the L3-35A enzyme previously reported and its thermally-stabilized mutants described so far (D02, C16, X01, X02A, X04, X07). Hence, we set out to design mutations to enhance the catalytic activity of X02A, the most thermally-stable mutant of our library (Table 1).

**Figure 2:**
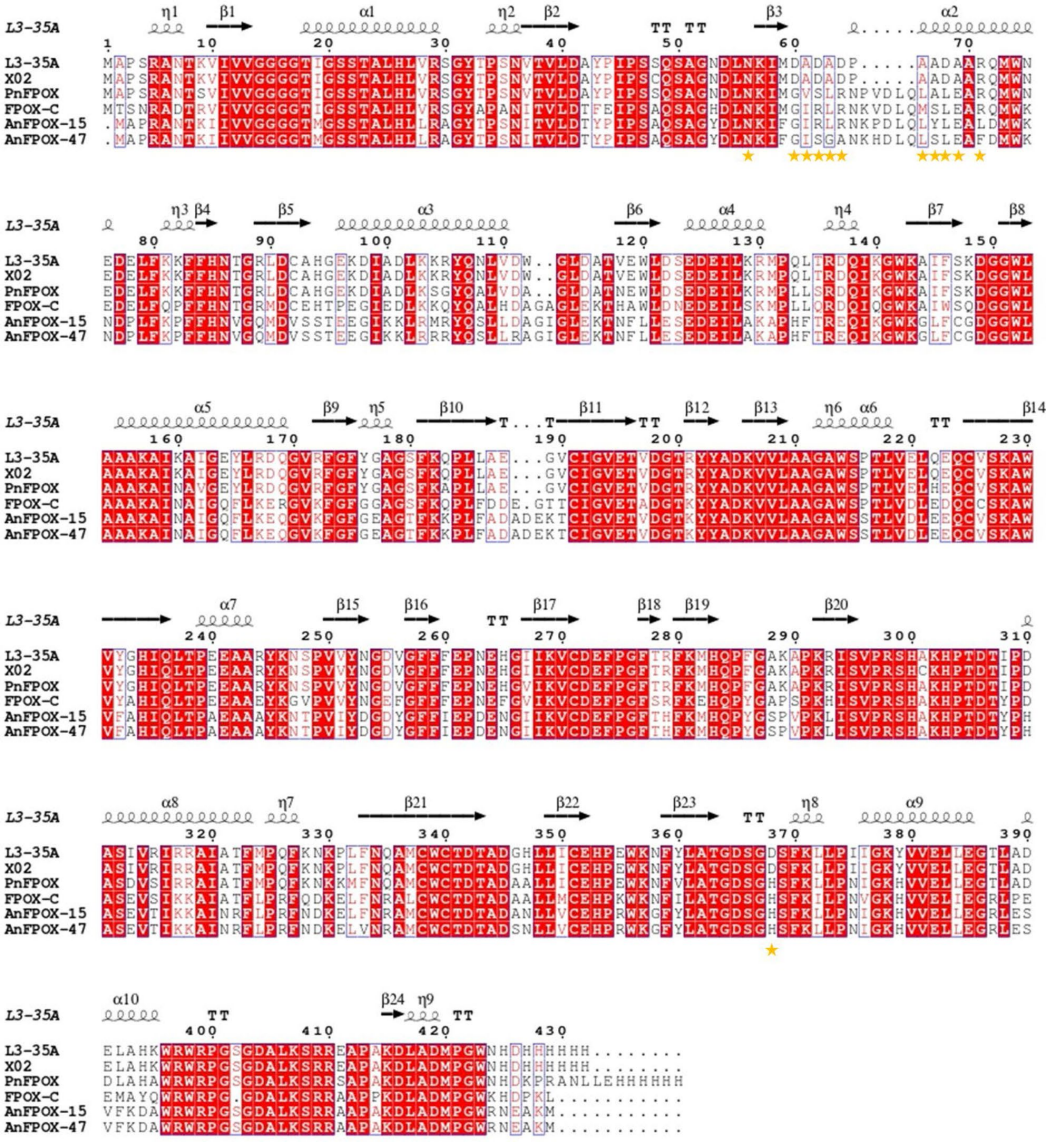
Sequence alignment of homologous enzymes used to design mutations for enzyme activity enhancement. The L3-35A and X02A enzymes were sequence- and structurally-aligned against the most active enzymes on the fructosyl-hexapeptide F6P that are available in the literature, namely PnFPOX and FPOX-C^6^, AnFPOX-15 and AnFPOX-47^24^. Yellow stars represent positions considered for mutagenesis.

To improve the catalytic activity of the engineered enzymes, the most promising variants resulting from the stabilization steps were sequence- and structurally-aligned with the most active enzymes against the fructosyl-hexapeptide F6P that are available in the literature, namely PnFPOX, FPOX-C and related variants^6^ and AnFPOX and related variants^24^. Specifically, tunnel-lining residues associated with improvements in the catalytic activity were selected to be introduced in the engineered enzyme (Fig. 2). Based on this comparison, we identified two designs in which enzyme activity could be partially restored. In the first design (X02B), four mutations were introduced in positions that are directly facing the tunnel in order to match the residues found in PnFPOX (D60G, A61V, D62S, D368H). In the second design (X02C), we also introduced the N56A mutation, which was shown to improve activity in the PnFPOX enzyme^6^, as well as a series of mutations in the loop 61–71. These mutations (A61I, D62S, A63G, D64A, A66L, A67S, D68L, A69E, R71F) were derived from the consensus sequence of PnFPOX and AnFPOX-47, the most active FPOX enzymes currently available.

**Figure 3:**
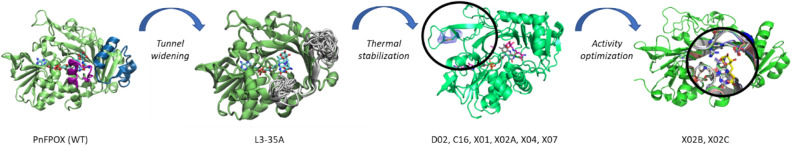
Schematic of the rational design strategy, showing the names of the relevant enzyme variants obtained at each stage.

In essence, by reintroducing conserved tunnel-lining residues in the X02A sequence, we generated the X02B and X02C mutants (Fig. 3) and we tested their activity toward the same panel of substrates that were tested with the parent enzyme (Table 2). It is worth noting that the X02B and X02C enzymes present limited activity toward the dipeptide (≈ 0.87 and 1.62 U/mg, respectively) compared to the WT (32.60 U/mg). However, while the WT significantly shows little, if any, ability to catalyze the oxidative reaction on the F6P hexapeptide (0.78 U/mg, which is equivalent to 2.4% of the activity on fVH), the X02B and X02C mutants feature significant activity also on the hexapeptide (0.18 and 0.43 U/mg, which are equivalent to 20.7% and 26.5% of the activity observed on the fVH). We measured the kinetic parameters (Table 3) for the WT and the latest engineered enzyme (X02C) against the fructosyl-valine substrate, which are found consistent with those reported in the literature for the enzyme family^7,10,27,29^. For the substrate fructosyl-hexapeptide, a comparison of the K_m_ of our mutant X02C (0.03 mM) with the wild type PnFPOX (0.81 mM) shows higher affinity for the substrate.

**Table 3:** Kinetic parameters of wild-type PnFPOX and the engineered X02C enzymes.

Enzyme	Substrate	K_m_ (mM)	V_max_ (mM/min)	k_cat_ (s^−1^)	k_cat_/K_m_ (mM^−1^ s^−1^)
PnFPOX	fV	5.93 ± 1.61	41.67 ± 8.34	99.33 ± 19.86	16.75
X02C	fV	0.94 ± 0.06	10.56 ± 0.56	20.83 ± 1.10	22.16
PnFPOX	F6P	0.81 ± 0.30	15.48 ± 1.20	36.83 ± 2.83	45.47
X02C	F6P	0.03 ± 0.01	0.26 ± 0.01	0.52 ± 0.02	17.22

To further compare our final design with similar FPOX, we compared it with a recently published engineered FPOX, named AnFPOX-47, which is reported to be the most active on intact glycated hemoglobin among the reported designs that present a wider tunnel^24^. The comparison shows that X02B and X02C have significantly higher activity than AnFPOX-47 toward F6P. Moreover, X02B and X02C feature a much higher T_m_ relative to all the other L3-35A mutants, i.e. 60.17 °C and 64.02 °C respectively (Table 1). Overall, within our panel of mutated enzymes the thermal stability of X02C increased significantly (more than 10 °C) with respect to the wild type PnFPOX. Interestingly, thermal stabilization also resulted in a significant increase in the production yield, the X02C variant featuring a nearly three-fold increase in expression yield with respect to the WT.

### Structural correlation analysis

To identify the possible molecular mechanism underlying the differences in enzymatic activity observed in our study, we determined the crystal structures of some of our best performing variants and we compared their overall fold as well as their tunnel and catalytic site. At the structural level, the tridimensional architecture of all mutants reported herein is maintained, confirming that our mutagenesis campaign did not impair the conformational stability of the enzyme. Indeed, when comparing the overall RMSD values of all the crystallized mutants to the wild type PnFPOX (PDB code 5T1E), the differences are all within a maximum of 1.8 Å (see Table 4).

**Table 4:** Calculated RMSDs between the WT enzyme PnFPOX and the different mutants, and tunnel geometry.

Enzyme	RMSD (Å)	Tunnel bottleneck radius (Å)	Tunnel length (Å)
PnFPOX	–	2.2	13.3
L3-35A	1.8	3.7	6.4
D02	1.4	2.9	11.7
X02A	1.7	3.1	9.4
X04	1.8	3.0	10.9
X02B	1.5	3.0	10.2

For selected enzymes, it was not possible to obtain the crystal structure.

**Figure 4:**
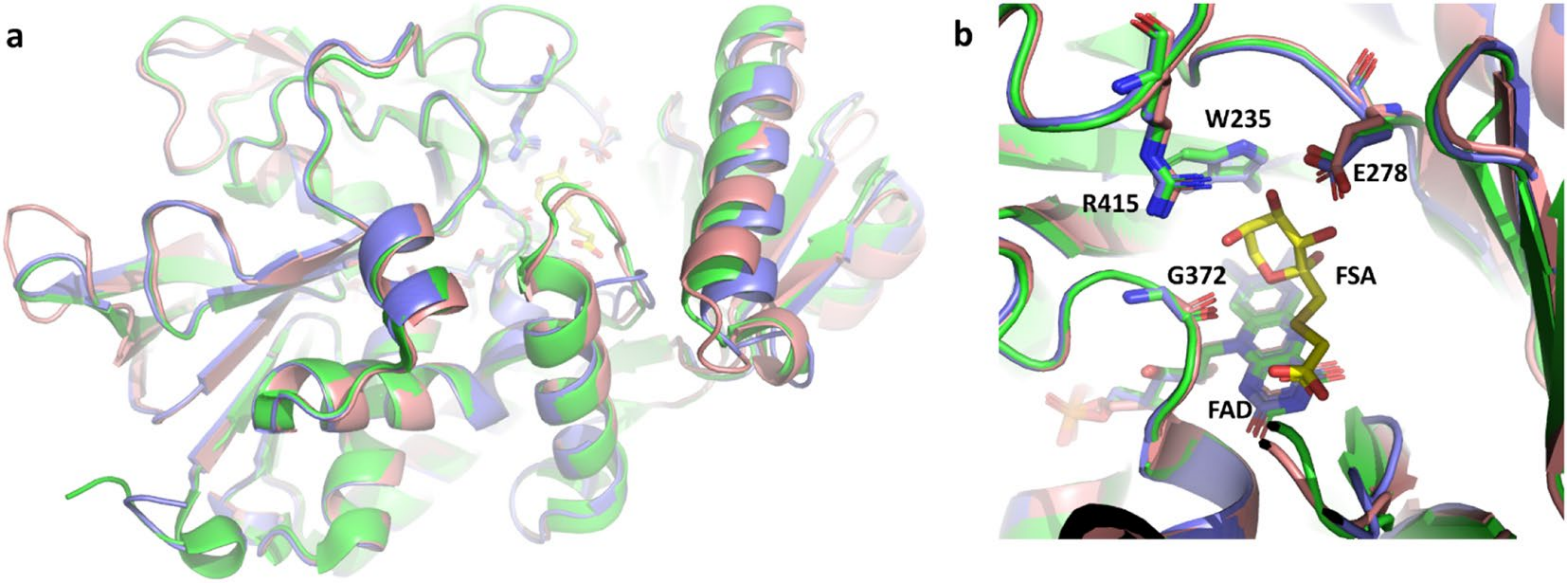
Structural comparison of relevant FPOX enzyme: PnFPOX (green), AnFPOX-47 (pink) and X02B (violet). The FSA inhibitor is depicted in yellow. The overall structural alignment shows no significant differences between the enzymes, except for the entrance tunnel (**a**). The position of the catalytic site is also highly conserved (**b**).

We then compared the residues in the catalytic site (W235, E278, G372, R415) of our mutants with those of the WT enzyme. These residues are highly conserved in all FPOX enzymes and bind to the sugar moiety of the substrates. We observed that the orientation of these residues is maintained (see Fig. 4), suggesting that the differences in the catalytic activity are not due to differences in the core catalytic site.

**Figure 5:**
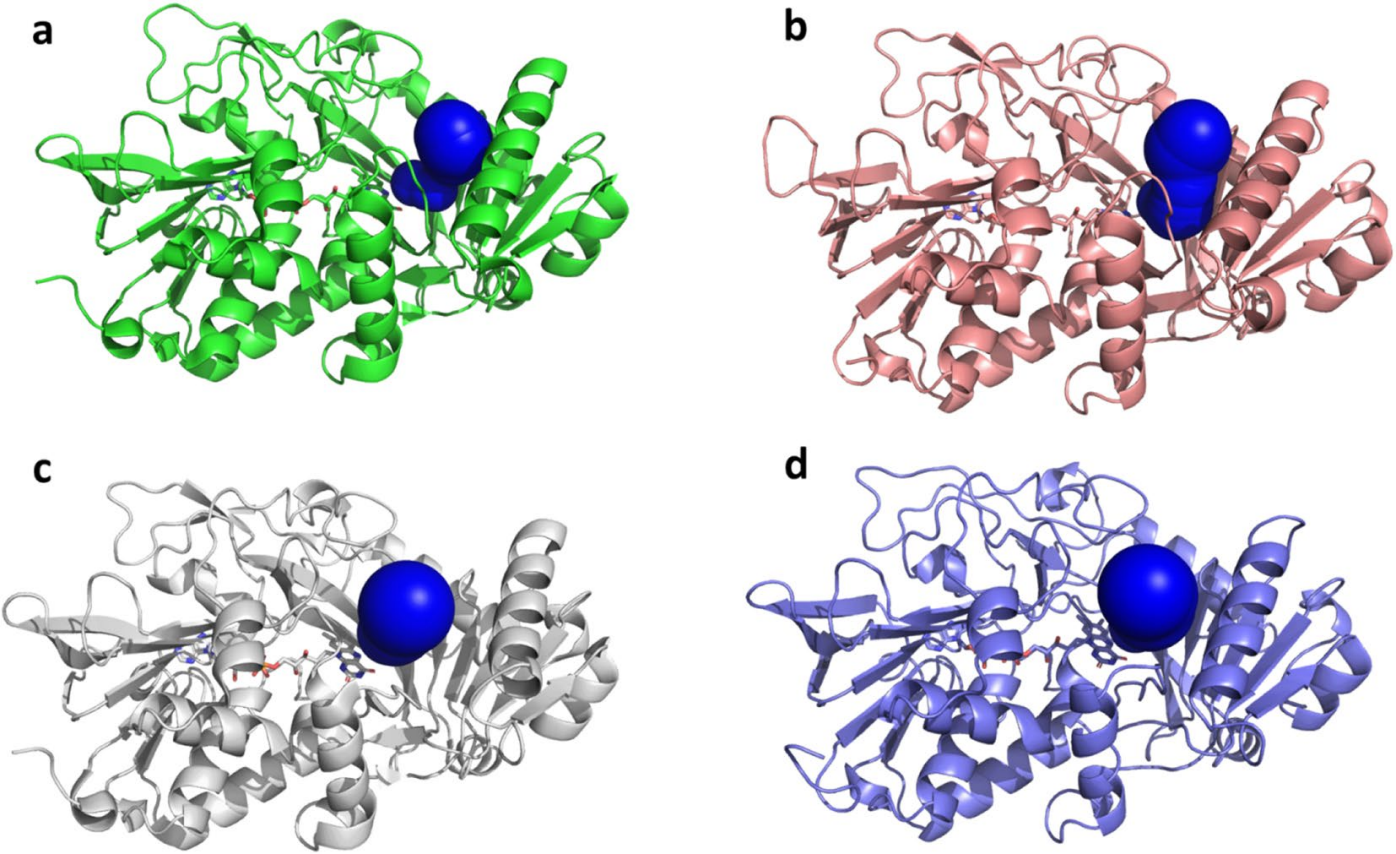
Comparison of the binding tunnel of PnFPOX (**a**), AnFPOX-47 (**b**), L3-35A (**c**) and X02B (**d**).

These results could be explained by the modification of the tunnel size and of its lining residues (see Fig. 5 and Table 4). In the WT, the small and long tunnel (bottleneck radius 2.2 Å, length 13.3 Å) allows greater stabilization of small substrates while limiting the entrance of larger substrates, hence explaining the marked decrease of activity when moving from glycated amino acids to the glycated hexapeptide. On the other hand, the AnFPOX-47 developed by Ogawa and coworkers^24^ presents a slightly larger tunnel (bottleneck radius of 2.7 Å) with comparable length (13.2 Å), which may explain its activity towards larger substrates. Our previously developed L3-35A enzyme variant^27^ exhibits a much wider and shorter tunnel (bottleneck radius of 3.7 Å and length of 6.4 Å) but shows limited activity on both small and large substrates, which could be due to the limited constraints of bound substrates due to the wider catalytic pocket, as well as to the removal of tunnel lining residues that contribute to the specific binding and stabilization of substrates. In an effort to revert the latter effect, we re-engineered the tunnel-lining residues in the X02B and X02C enzymes. The X02B enzyme features a wide and short tunnel (bottleneck radius of 3.0 Å and length of 10.2 Å), in between the WT and its parent L3-35A, while at the same time showing a similar activity on both small and larger substrates. Hence, the wider tunnel of X02B, while reducing the stabilization of small substrates (and, as a consequence, the catalytic activity towards them) allows larger substrates to access the catalytic site more easily, leading to comparable enzymatic activity.

## Conclusions

As part of an ongoing investigation aimed at engineering an FPOX enzyme to provide it with both enhanced thermostability and a wider access tunnel to the catalytic site, we set out to design a number of novel FPOX mutants starting from our previously characterized mutant L3-35A of PnFPOX, which featured a much larger active site relative to the wild type enzyme. To increase enzyme thermostability, we tested several strategies, namely introducing mutations to either improve the RMSF of the protein, increase the number of salt bridges or form single disulfide bonds. Our thermostability findings align with the expectation that the improved RMSF, salt bridges formation, and disulfide bonds formation approaches can all effectively improve the thermal stability of enzymes, albeit to a different extent. In particular, we have shown that the introduction of a disulfide bond allowed us to produce a PnFPOX variant that shows remarkably higher thermal stability compared to the other mutants. Furthermore, the catalytic activity, which was significantly lost during the design process, was reintroduced by engineering ligand-binding and tunnel-lining position, which led to a new, thermally stabilized and active FPOX enzyme that features a wider access tunnel to the catalytic pocket. Ongoing efforts are aimed at testing the enzymes on whole glycated proteins (i.e., albumin and hemoglobin) to test whether the wider access tunnel of X02C, together with the restored catalytic activity, can actually provide catalytic activity directly on whole proteins. This may represent a significant step toward the use of these enzymes on larger substrates and on full proteins, thus possibly allowing the development of in vitro HbA1c diagnostic devices based on a direct enzymatic oxidation system. Indeed, there is a rapidly growing demand for cheap, efficient and rapid diabetes monitoring tests. This could be met by developing enzymatic assays for glycated hemoglobin and albumin that do not require a preliminary digestion of the proteins. The elimination of this step would provide several advantages to the test, such as the shortening of its measuring times, a simplification of its overall procedure and a substantial reduction of its costs.

From the perspective of protein engineering, the use of rational design methods like the one described in this work offers a promising strategy for the design of thermostable enzymes with improved performance in various industrial and biomedical applications. This study also highlights the potential of computational methods and targeted mutations to improve the activity and substrate range of enzymes, and it paves the way for the design of thermostable enzymes with improved performance toward different targets and applications. We believe that this rational approach can accelerate the production and the use of biocatalysts in different industrial sectors, thus in turn allowing the improvement of chemical processes through the application of green chemistry concepts.

## Experimental procedures

### Molecular dynamics simulations

To identify the most suitable regions for structural modifications in the L3-35A enzyme, we ran 100 ns MD simulations following previously adopted protocols^23,27,30^. Specifically, we used the AMBER19SB force field^31^ for protein, water (TIP3P) and ions, while we used the general amber force field (GAFF)^32^ for modeling the FAD cofactor. We solvated the molecular model with a 15 Å pad of TIP3P water and we introduced counter ions to neutralize the system charge, resulting in a final simulation box of ≈ 50.000 atoms. Hydrogen mass repartitioning was applied to allow a time step of 4 fs^33^. The system was subjected to 1000 energy minimization steps and it was equilibrated for 100 ps at a pressure of 1 atm and at a temperature of 300 K with NAMD software^34^, using a non-bonded cut-off of 12 Å, rigid bonds and particle-mesh Ewald long-range electrostatics. During the equilibration simulation, the Cα atoms of the protein were restrained by a 10 kcal mol^−1^ Å^−2^ spring constant. The 100 ns production runs were performed using a NVT ensemble whereby all the parameters (non-bonded cut-off, and PME) were the same as in the equilibration phase. Root mean square deviations (RMSD) were monitored after each MD run to assess structural convergence, while root mean square fluctuations (RMSF) were calculated to identify unstable regions for subsequent design. The tunnel dimension was analyzed with CAVER 3.0.3 software^35^ using a probe radius of 1.4 Å, a shell depth of 5 Å, and a shell radius of 2 Å.

### Design and selection of stabilizing mutations

The identification of stabilizing mutations was performed using five different tools. The design focused on the most unstable region of the enzyme as identified in the previous step, with the exclusion of the catalytic amino acids, the amino acids interacting with the FAD cofactor, and the residues lining the tunnel. The first design was performed using the Rosetta Supercharge tool^36^. This tool was employed to reengineer the protein surface with amino acids that present a high net charge, which is reported to prevent aggregation of partially unfolded states. The second design was performed using the Rosetta Point Mutant “pmut” scan application^37^, which helped to identify stabilizing single point mutations. In the third approach, the *pmut* tool was used to identify stabilizing double point mutations, where only pair of residues that are close enough to interact with each other are tested (i.e., the distance of at least one of their atoms being closer than 4.5 Å). In the fourth approach, the PROSS web server^38^ was used to generate 9 different enzyme designs with an incremental number of mutations. Disulfide bond engineering was performed by employing the disulfide-by-design web server^39^. For each of the 5 design strategies, the best 20 enzyme variants based on the internal scoring were retained (except for PROSS design, where all 9 variants were retained, and for disulfide design, where 8 variants were retained) for further analysis with MD simulations. Overall, library generation and ranking using the five different methods resulted in the selection of 77 different enzyme variants. Each model was simulated for 100 ns, as described for the reference L3-35A enzyme, and the best 6 variants in terms of RMSF stabilization^27,30^ were selected for experimental validation.

### Protein expression and purification

The DNA sequences of the different FPOX variants were cloned in either pET15(b) or pET17(b) expression vectors (Novagen), to generate C-terminal 6His-tagged proteins. Reference 5T1E and L3_35 DNA constructs, as well as the D02 and the C16 variants, were transformed and expressed in E. coli BL21 Star (DE3) cells (Invitrogen). Variants featuring cysteine mutations to allow disulfide bond formation (X02A, X04, X07, X02B, X02C) were transformed in SHuffle T7 E. coli cells (New England Biolabs). For each variant, 20 mL of an overnight culture was inoculated into 2L of Luria–Bertani (LB) broth supplemented with 100 mg/L ampicillin and grown at 37 °C until OD_600_ = 0.6. Protein expression was inducted using isopropyl 1‐thio‐β‐d‐galactopyranoside (IPTG) at a final concentration of either 0.4 mM or 0.1 mM, followed by overnight culture at either 25 °C or 18 °C respectively, with shacking speed of 220 rpm. Cells were collected by centrifugation and resuspended in lysis buffer (50 mM Tris‐HCl pH 7.4, 150 mM NaCl, 0.5 mM FAD, and 5% glycerol), supplemented with 0.2 mM protease phenylmethylsulfonyl fluoride (PMSF) and DNAse and then lysed by sonication on ice. After centrifugation, the soluble fraction of the cell lysate was passed through a 0.45-micron filter and then loaded onto a HisTrap HP 5 mL (GE Healthcare) column equilibrated with buffer A (50 mM Tris‐HCl pH 7.4, 150 mM NaCl, 5% glycerol, 20 mM imidazole). The Ni affinity column was washed using ten column volumes of buffer A, then the bound C‐terminal His‐tagged enzyme was eluted with the same buffer supplemented with 400 mM imidazole. The eluted fraction was loaded onto a Hiprep 26/60 Sephacryl S-100 size exclusion column (GE Healthcare) pre‐equilibrated with buffer C (50 mM Tris‐HCl pH 7.4, 150 mM NaCl, 5% glycerol). The desired protein was concentrated using an Amicon 20 centrifugal filter with a molecular cutoff of 10 KDa to ≈ 2 mg/mL, and stored at − 80 °C. Protein concentration was assessed using a NanoDrop (Thermo Scientific). Sample purity was evaluated by 12% SDS-PAGE.

### Protein crystallization

Crystals of different mutants (D02, X02A, X04, X07, X02B) were grown at room temperature using the vapor diffusion method by mixing a 2 μL drop of a 10–15 mg/mL protein sample with a 2 μL drop of a solution containing 0.1 M MES monohydrate pH 6.5, 16–26% of different Polyethylene glycol (PEG) including 20 K (for D02, X02A and X02B) and 8 K (for X04). Crystals, which appeared after 1–4 days, were frozen using 25% (v/v) glycerol as cryoprotectant prior to X-ray diffraction data collection.

### Data collection and processing

For the FPOX D02 crystals, diffraction data were collected using a Dectris PILATUS3 6 M detector and a radiation of λ = 0.918 Å on Bessy beamline 14.1 of the Deutsches Elektronen- Synchrotron (BESY II Light Source), Berlin, Germany. Reflection-image processing was performed using DIALS^40^ and AIMLESS^41^ from the CCP4 suite^42^. For FPOX X02A and X04, X-ray diffraction data were collected using radiation of λ = 1.0 Å and a PSI PILATUS 6 M detector on the X06DA beamline at Swiss Light Source (SLS). Data was processed using autoproc. For X02B, diffraction data were collected using a DECTRIS EIGER2 XE 16 M detector and a radiation of λ = 0.9537 Å on the I04 beamline at Diamond Light Source (Oxfordshire, United Kingdom). Data was processed using the autoproc package. Data collection and refinement statistics are shown in Table 5.

**Table 5:** Diffraction data collection and refinement statistics. Statistics for the highest-resolution shell are shown in parentheses.

	D02	X02A	X02B	X04
PDB code	8BLZ	8BLX	8BJY	8BMU
Wavelength (Å)	0.918	1.000	0.9537	1.000
Space group	C 2 2 2_1_	C 2 2 2_1_	P 2_1_ 2_1_ 2_1_	P 4_2_ 2_1_ 2
Unit cell (Å)	a = 91.10, b = 129.49, c = 86.78	a = 90.52, b = 130.26, c = 87.62	a = 63.11, b = 88.49, c = 90.94	a = 90.24, b = 90.24, c = 131.44
Resolution range (Å)	64.75–1.70 (1.73–1.70)	56.68–1.71 (1.74–1.71)	63.42–1.48 (1.50–1.48)	74.39–1.645 (1.673–1.645)
Total reflections	707,191 (38,534)	641,243 (29,830)	1,053,583 (48,251)	1,012,704 (25,719)
Unique reflections	55,830 (2946)	55,592 (2685)	82,805 (4255)	62,950 (2416)
Multiplicity	12.70 (13.10)	11.5 (11.10)	12.7 (11.3)	16.1 (10.6)
Completeness (%)	98.70 (99.30)	98.9 (96.5)	96.0 (100.0)	94.7 (74.1)
mean(I)/sig(I)	12.60 (2.20)	12.7 (1.0)	16.7 (0.4)	20.5 (1.1)
Wilson B-factor	12.08	21.4	27.04	11.12
Rmerge	0.17 (1.40)	0.152 (2.416)	0.067 (4.526)	0.106 (2.013)
Rmeas	0.17 (1.45)	0.159 (2.532)	0.070 (4.743)	0.110 (2.104)
Rpim	0.05 (0.39)	0.046 (0.743)	0.020 (1.406)	0.026 (0.589)
CC1/2 (%)	1.00 (0.75)	0.998 (0.461)	1.000 (0.419)	0.999 (0.458)
Reflections in refinement	55,772 (3879)	55,537 (5360)	80,561 (7099)	60,833 (939)
Reflections in free set	2729 (209)	2755 (264)	3995 (332)	3043 (44)
Rwork	0.177	0.1812	0.19	0.1766
Rfree	0.199	0.2103	0.215	0.2009
RMSD bonds (Å)	0.0144	0.011	0.012	0.012
RMSD angles (Å)	2.214	1.74	1.75	1.84
Ramachandran favoured (%)	97.2	95.74	96.71	95.52
Ramachandran allowed (%)	2.8	3.78	3.06	4.01
Ramachandran outliers (%)	0	0.47	0.24	0.47
Rotamer outliers (%)	1.1	3.68	0.85	2.53
Clash score	1.2	5.38	4.28	6.23
Overall number of atoms (non-H)	3745	3788	3758	3917
In macromolecules	3350	3354	3364	3376
In ligands	99	119	65	113
In solvent	296	315	329	428
Average B-factor (Å^2^)	21.7	30.07	30.39	22.03
For macromolecules	21	29.46	29.53	20.32
For ligands	23.9	37.65	32.17	34.43
For solvent	28.3	33.68	38.91	32.23

### Structure determination and refinement

For all four structures, the initial phases were obtained by molecular replacement using Phaser^43^ and the atomic coordinates of the L3-35A FPOX mutant (PDB code 6Y4J^27^) as a search model. Refinement was performed by alternating rounds of REFMAC5^44^ and manual adjustments in Coot^42^. Water molecules were added both manually and automatically using the Coot_refine tool from the CCP4 cloud package^45^.

### Thermal shift assay

The melting temperature of each variant was measured using differential scanning fluorimetry (DSF) with Sypro-Orange dye (TermoFisher Scientific) on an Applied Biosystems 7500 Real-Time PCR system (TermoFisher Scientific). For each mutant, 12.5 μL of enzyme at the starting concentration of 10 μM was added to 12.5 μL of a 10× Sypro-Orange dye solution diluted from a 5000× stock in the same buffer that was used for the protein: 50 mM Tris‐HCl pH 7.4, 150 mM NaCl, 5% glycerol) to reach the final volume of 25 μL, a 5× final concentration of the dye and a 5 µM concentration of the enzyme. All the Tm measurements were performed in 4 replicates. Fluorescence was monitored during the thermal denaturation occurring to the protein upon increasing the temperature from 15 to 95.3 °C.

### Circular dichroism

Circular dichroism (CD) measurements were performed on a Jasco J-1500 spectrophotometer at 20 °C. CD was performed to detect the secondary structure of different variants and to measure the thermostability of the enzyme. The spectra were the average of five scans, recorded using a 1.0 mm path length quartz cuvette on 5 μM samples of the different FPOX variants in a 50 mM Tris pH 8.0, 150 mM NaCl, 5% glycerol buffer solution. Thermal denaturation curves were measured in 1.0 mm path length cuvettes closed with a parafilm on 5 µM samples. The increasing temperature-induced denaturation at a rate of 1 °C/min from 5 to 95 °C and the ellipticity at 222 nm were recorded at 0.2 °C intervals using a 1-nm bandwidth and a response of 10 s. Using the Boltzmann function, the midpoints of the thermal-denaturation curves (T_m_) were determined by fitting the data to a sigmoidal transition curve. The secondary structure of different variants was measured at 200–250 nm wavelengths at 20 °C. Mean residue ellipticity [θ]MR was calculated and plotted versus wavelength. Each spectrum was determined as the average of three scans. The CD spectra of all the enzymes described here are shown in Figs. S1–S7 in the Supplementary Information.

### Enzymatic activity

The enzymatic activity of all variants was assessed at room temperature by measuring the amount of glucosone released from the substrate over time, as described previously^27^. The increase in absorbance at 322 nm (glucosone ε_322_ = 149.25 M^−1^ cm^−1^) was monitored in an Infinite M1000 (Tecan) at 25 °C. The 200 μL reaction mixture contained 20 mM Tris HCl pH 7.4, 20 mM o-phenylenediamine, 2 mM fructosyl-lysine, or fructosyl-valine or dipeptide (fructosyl-valine histidine) or hexapeptide (1-Deoxyfructosyl-Val-His-Leu-Thr-Pro-Glu) as a substrate. After 1 min of pre-incubation, the reaction was started by adding the enzyme at a final concentration of 0.04–1 mg/mL depending on the activity of the variant. One unit (U) is defined as the amount of enzyme required to produce 1 µmol of glucosone per minute, and specific activity is expressed as U/mg of the enzyme. Enzyme kinetic constants (Vmax, Km, kcat and kcat/Km) were determined using different substrate concentration in the assay (fructosyl-valine from 0.05 to 10 mM, fructosyl hexapeptide from 0.05 to 1 mM). Data points are the result of two independent experiments and the estimation of Micheaelis-Menten parameters was derived by Lineweaver-Burk plot fitting.

### Supplementary Information

Supplementary Figures.

## Data Availability

The final crystallographic coordinates of the crystal structures shown here are available in the RCSB PDB (accession codes: 8BJY, 8BLZ, 8BLX, 8BMU).
