# Supplementary material for: Tailoring FPOX enzymes for enhanced stability and expanded substrate recognition
Source: Sci Rep. 2023 Oct 30;13:18610. doi: 10.1038/s41598-023-45428-1 (PMC10616090; doi:10.1038/s41598-023-45428-1)
Supplement: Supplementary file 1 — Supplementary Figures. [file 41598_2023_45428_MOESM1_ESM.docx]

**Supplementary Data**

**Tailoring FPOX Enzymes for Enhanced Stability and Expanded Substrate Recognition**

Hajar Estiri^1,≠^, Shapla Bhattacharya^1,2≠^, Jhon Alexander Rodriguez Buitrago^1^, Rossella Castagna^1,3^, Linda Legzdiņa^1^, Giorgia Casucci^1^, Andrea Ricci^4^, Emilio Parisini^1,5,*^, Alfonso Gautieri^4,*^

^1^ Department of Biotechnology, Latvian Institute of Organic Synthesis, Aizkraukles 21, LV-1006, Riga (Latvia)

^2^ Faculty of Materials Science and Applied Chemistry, Riga Technical University, Paula Valdena 3, LV-1048 Riga (Latvia)

^3^ Dipartimento di Chimica, Materiali e Ingegneria Chimica “Giulio Natta”, Politecnico di Milano, piazza L. da Vinci 32, 20133 Milano (Italy)

^4^ Biomolecular Engineering Lab, Dipartimento di Elettronica, Informazione e Bioingegneria, Politecnico di Milano, Piazza Leonardo da Vinci 32, 20133 Milano (Italy)

^5^ Department of Chemistry “G. Ciamician”, University of Bologna, Via Selmi 2, 40126 Bologna (Italy)

≠ These authors contributed equally to this work

* To whom correspondence should be addressed: Emilio Parisini ([emilio.parisini@osi.lv](mailto:emilio.parisini@osi.lv)), Alfonso Gautieri (alfonso.gautieri@polimi.it)

**Keywords**

*Protein engineering, protein stability, enzyme mutation*, *access tunnel, fructosyl peptide oxidase*, *molecular modeling, biosensor, diabetes*


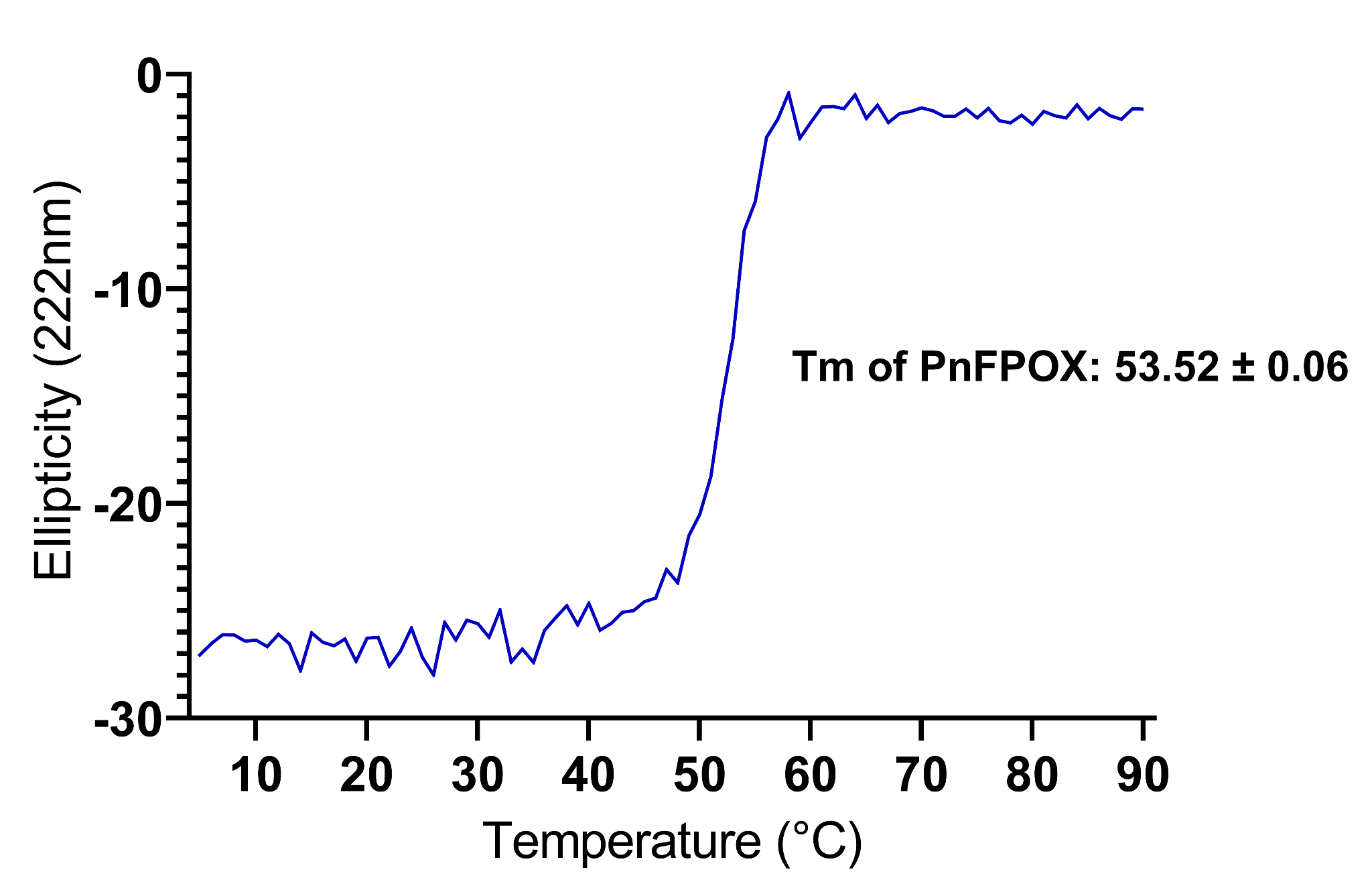


Figure S1. Tm of PnFPOX as measured by circular dichroism at 222nm. The determination of the midpoints of the thermal-denaturation curves (Tm) involved fitting the data to a sigmoidal transition curve using the Boltzmann function.


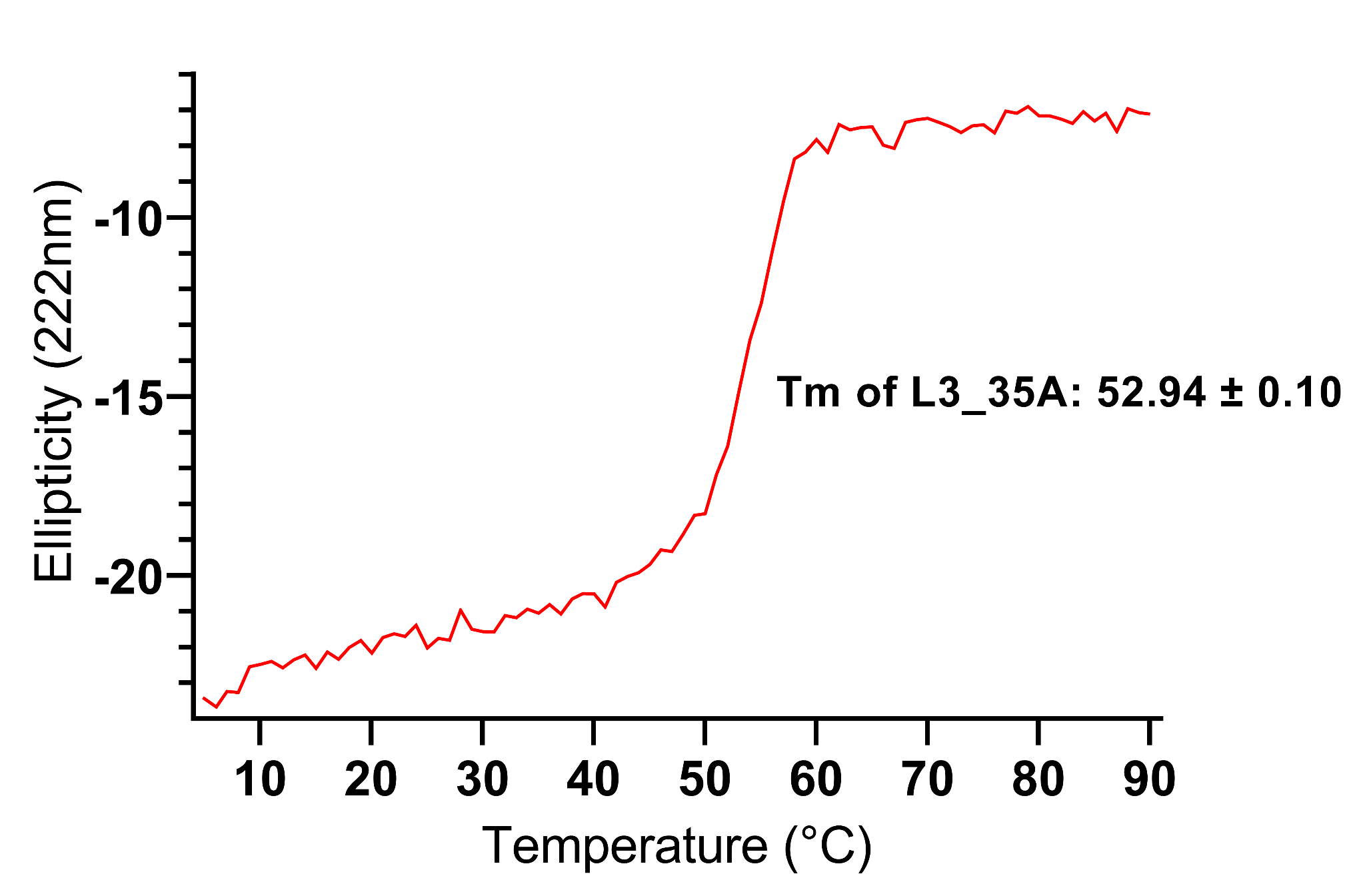


Figure S2. Tm of L3_35A as measured by circular dichroism at 222 nm from 5 °C to 95 °C.


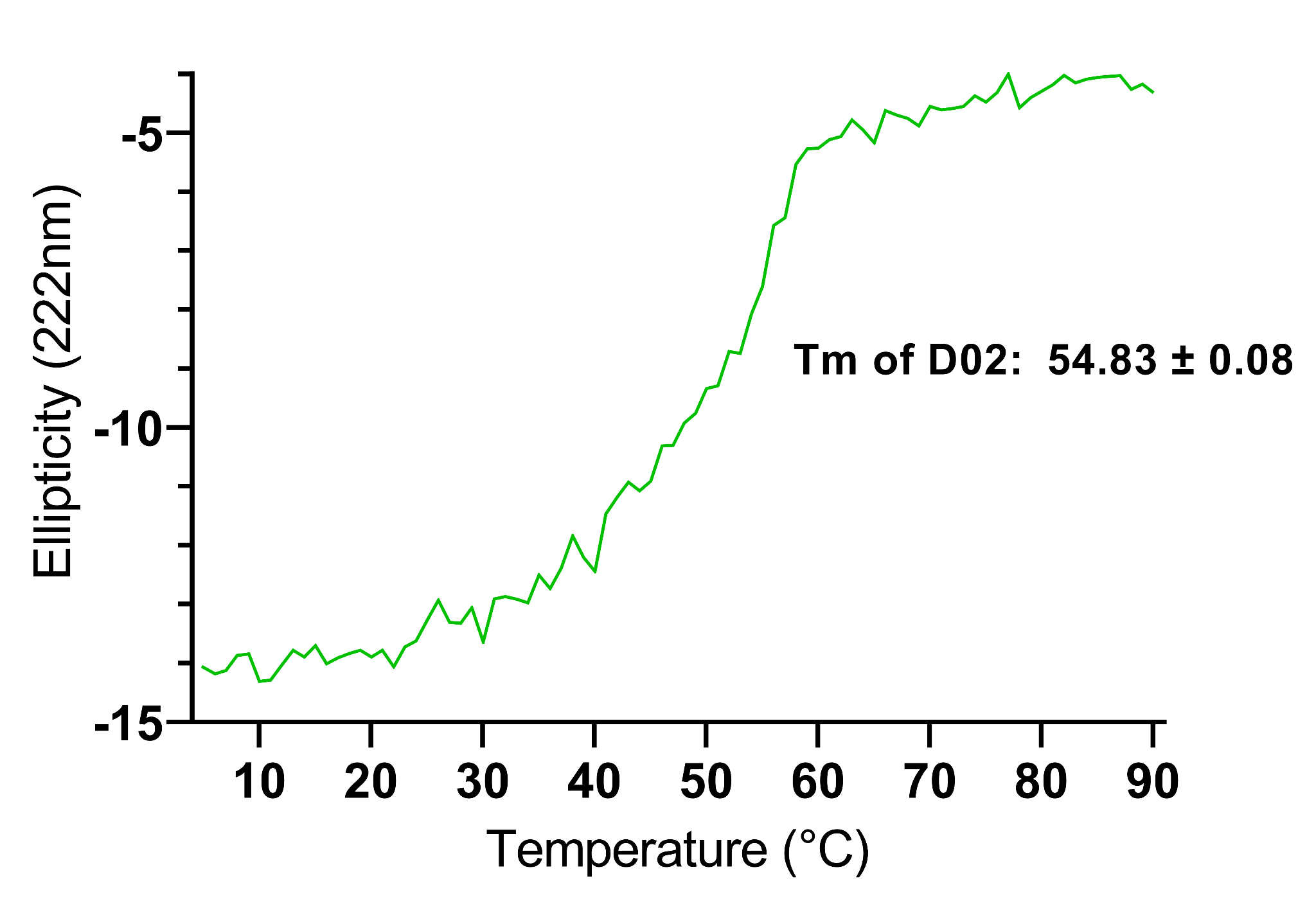


Figure S3. Tm of D02 as measured by circular dichroism at 222 nm from 5 °C to 95 °C.


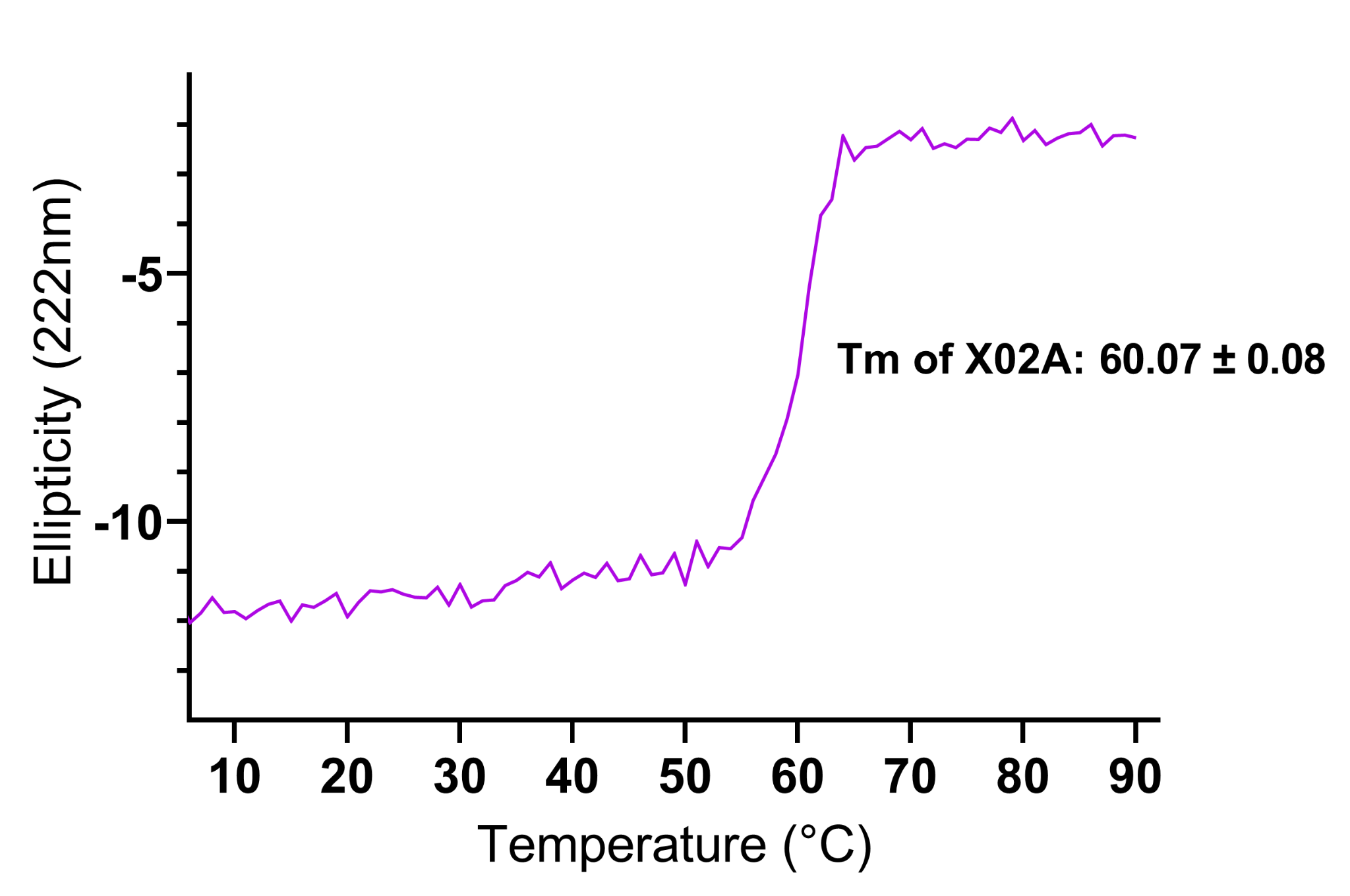


Figure S4. Tm of X02A as measured by circular dichroism at 222 nm from 5 °C to 95 °C.


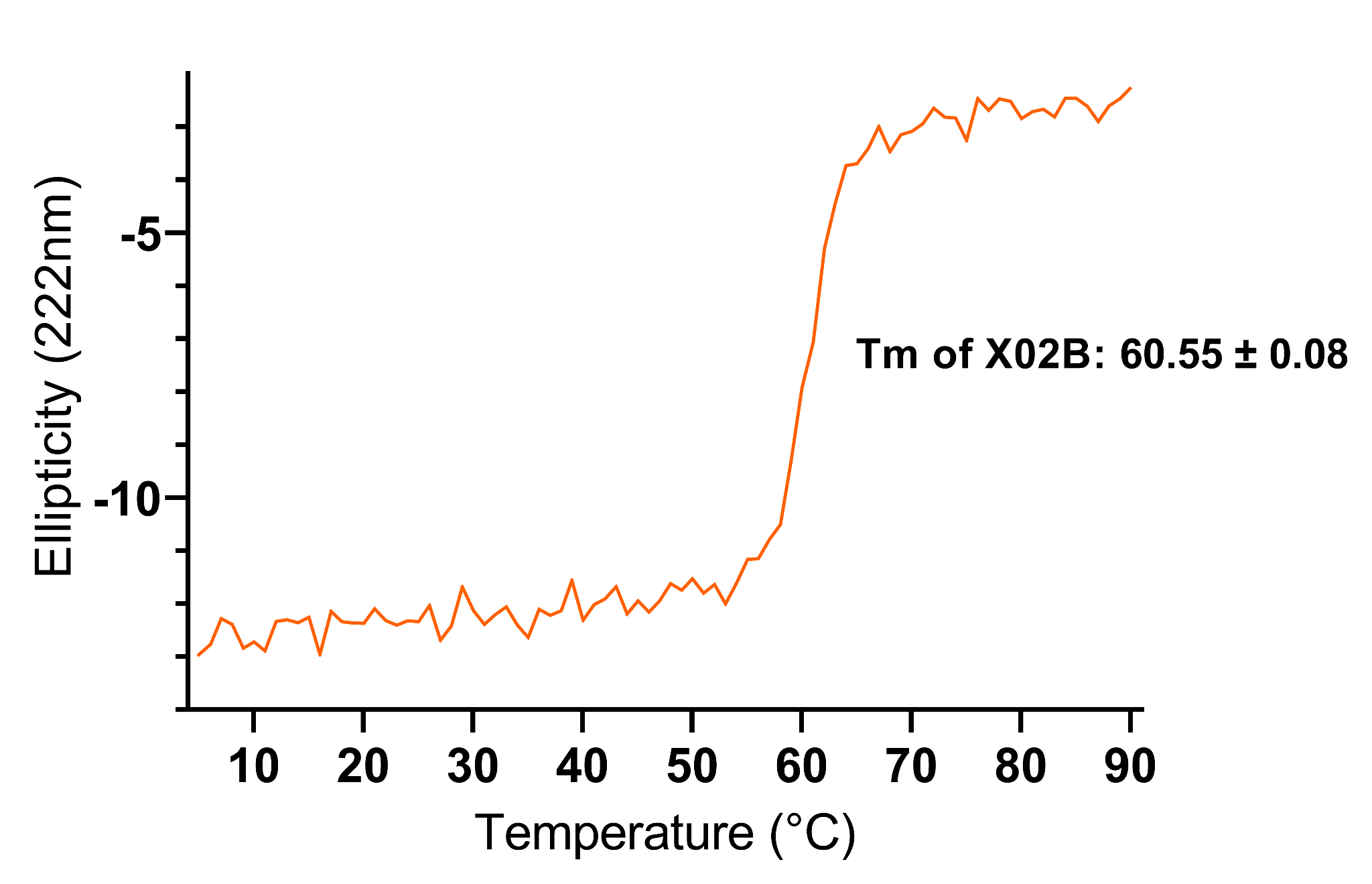


Figure S5. Tm of X02B as measured by circular dichroism at 222 nm from 5 °C to 95 °C.


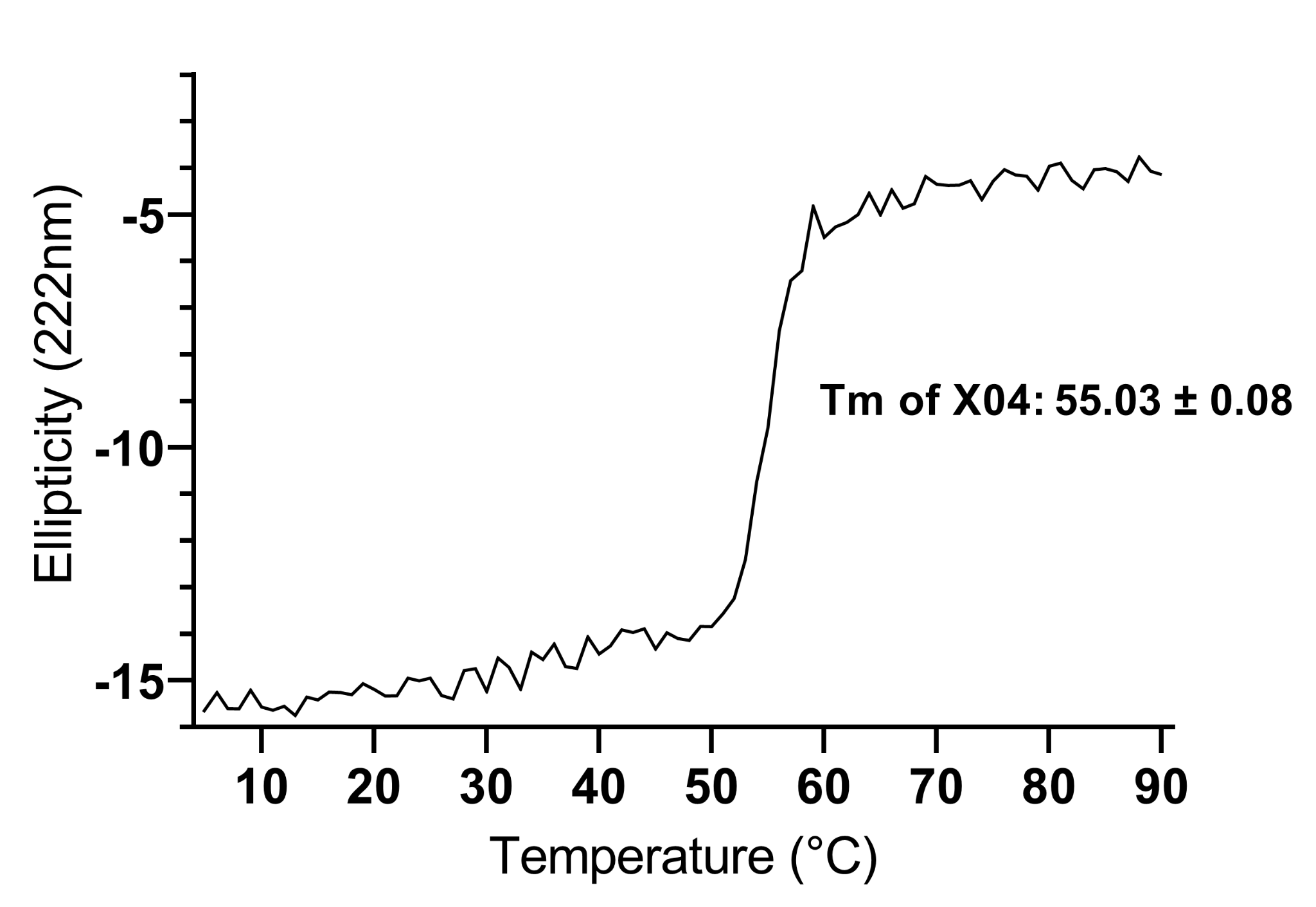


Figure S6. Tm of X04 as measured by circular dichroism at 222 nm from 5 °C to 95 °C.


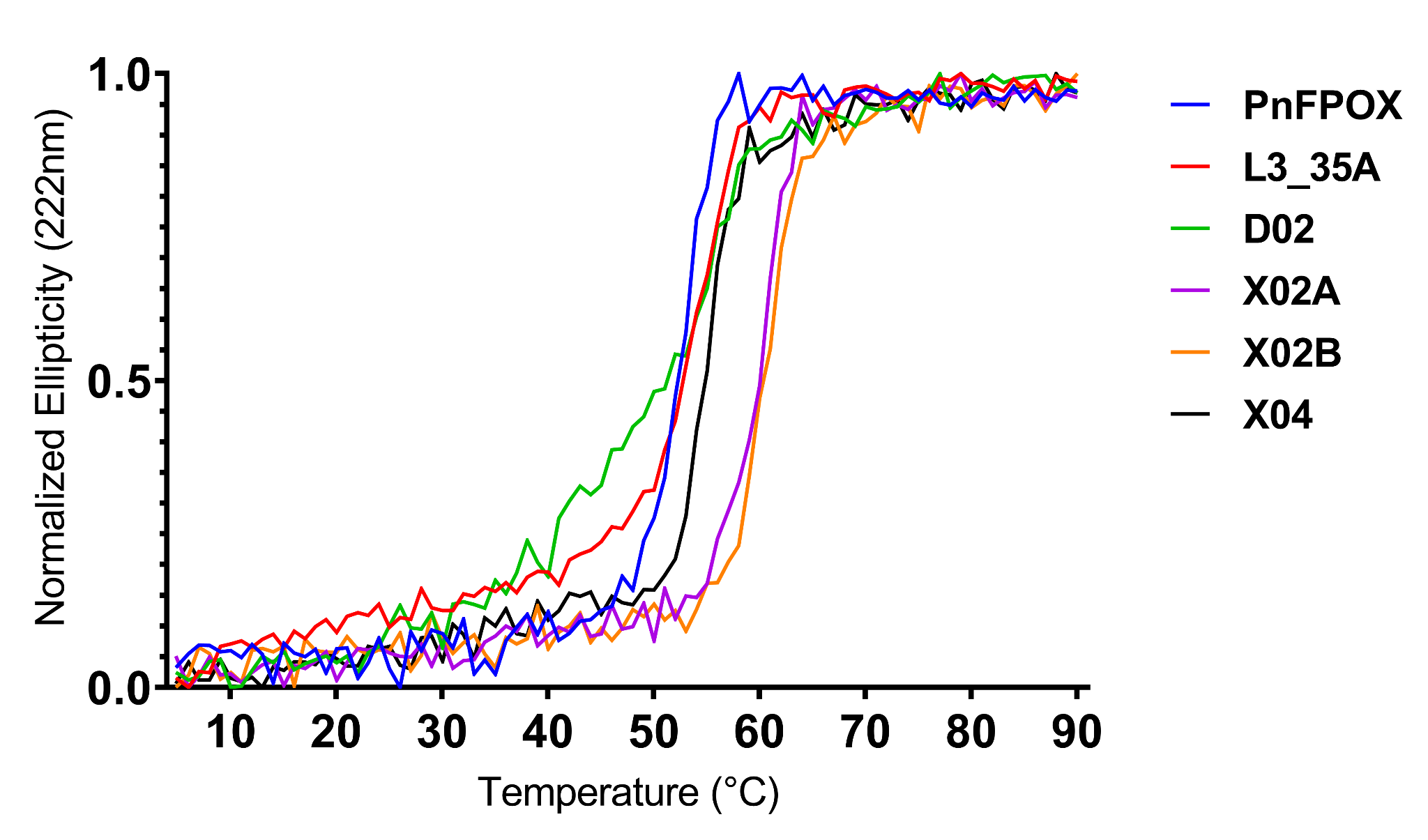


Figure S7. Comparison of the Tm values of the different FPOX variants as measured by circular dichroism at 222 nm from 5 °C to 95 °C.

Table S1. Expression yield of enzymes

| **Enzyme** | **Expression**  **yield (mg/L)** | **Expression Temperature(°C)** | **Expression**  **Cells** |
| --- | --- | --- | --- |
| PnFPOX | 10 | 25 | BL21 Star (DE3) |
| L3-35A | 30 | 25 | BL21 Star (DE3) |
| D02 | 15 | 25 | BL21 Star (DE3) |
| C16 | 12 | 25 | BL21 Star (DE3) |
| X01 | 5 | 18 | SHuffle T7 E |
| X02A | 20 | 18 | SHuffle T7 E |
| X04 | 10 | 18 | SHuffle T7 E |
| X07 | 6 | 18 | SHuffle T7 E |
| X02B | 16 | 18 | SHuffle T7 E |
| X02C | 29 | 18 | SHuffle T7 E |


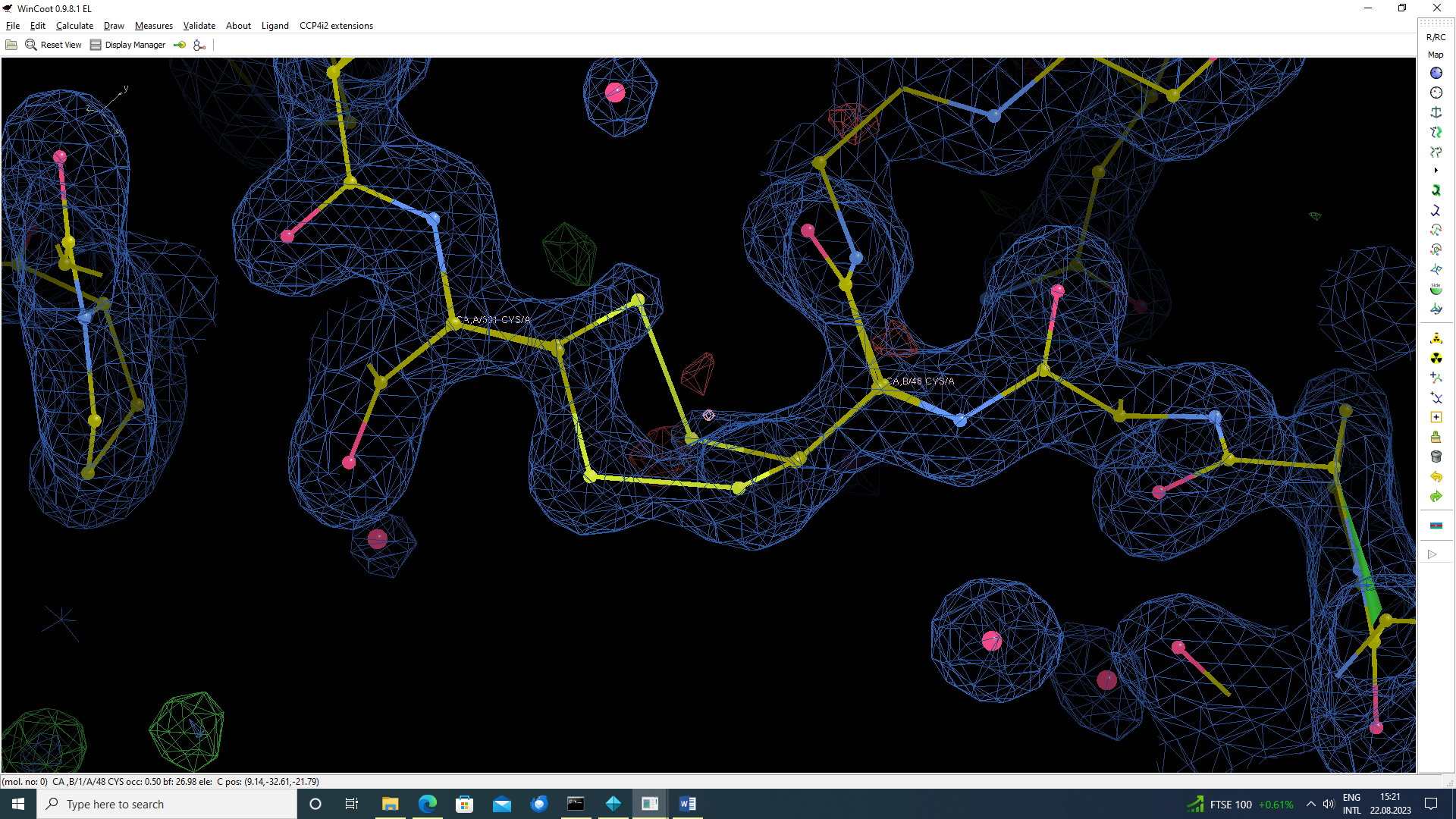


Cys301

Cys48

Figure S8. Crystal structure of X02B (PDB: 8BJY) showing the formation of disulphide bond between Cysteine 48 and 301


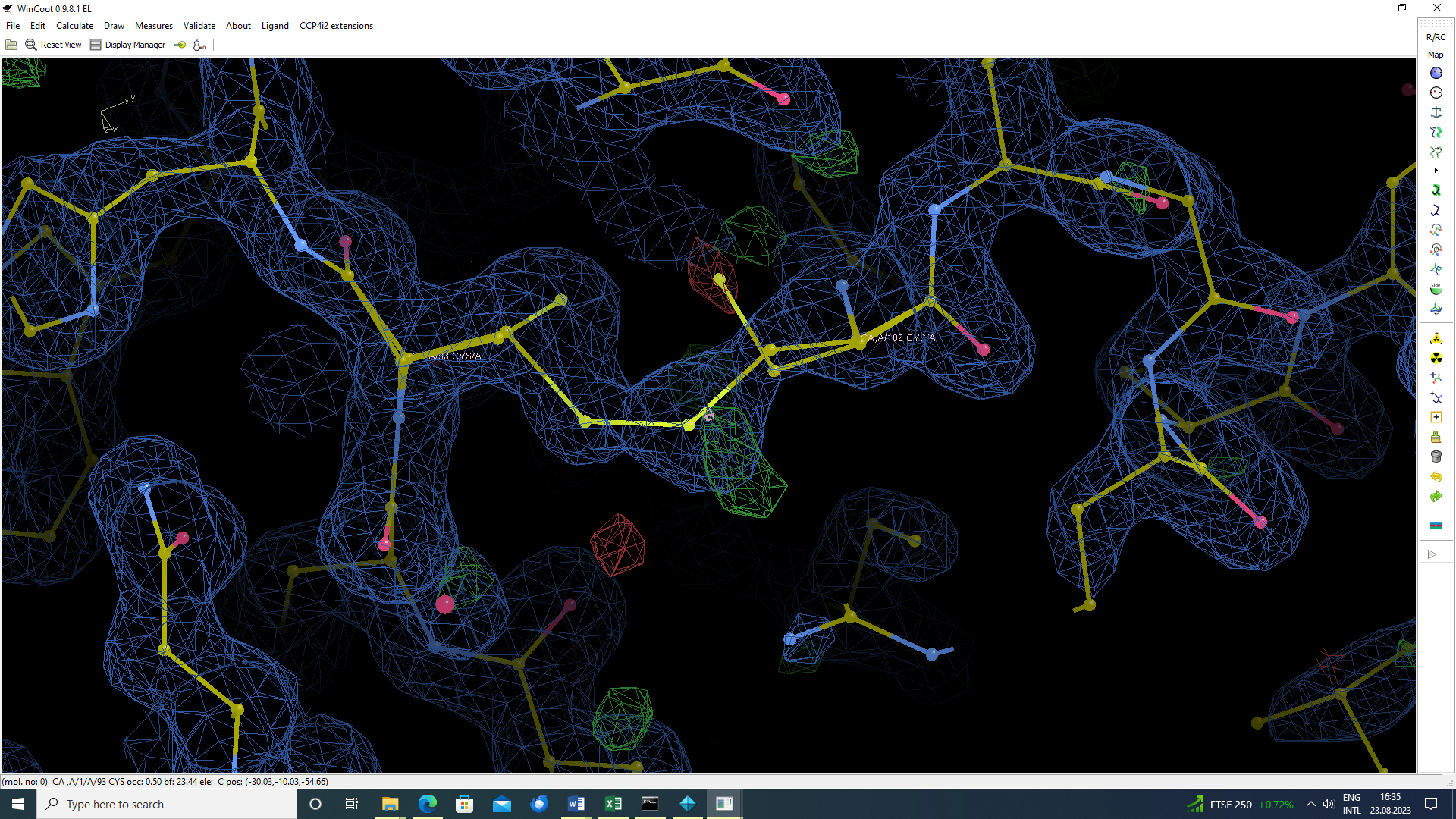


Cys93

Cys102

Figure S9. Crystal structure of X04 (PDB: 8BMU) showing the formation of disulphide bond between Cysteine 93 and 102


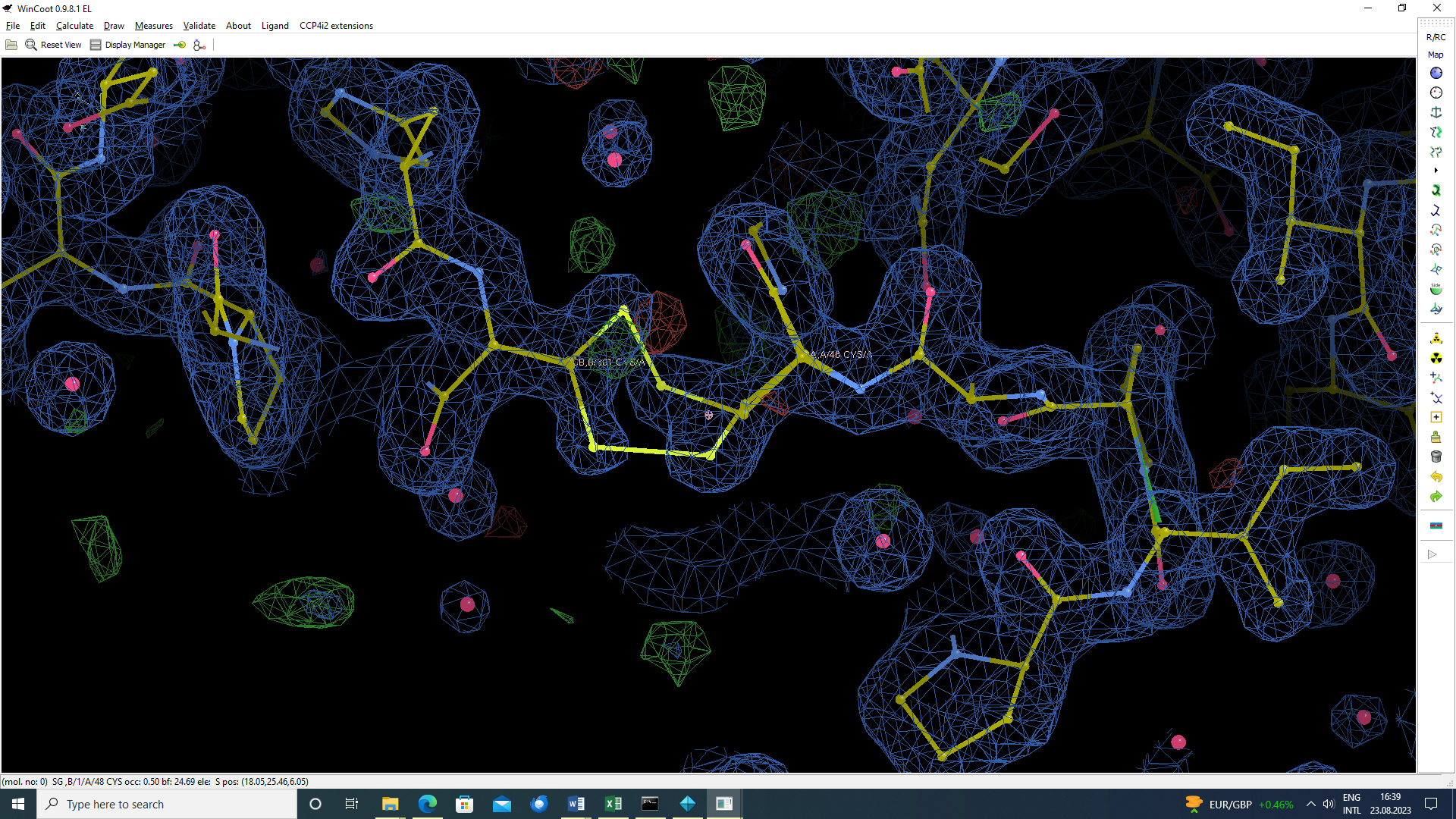


Cys301

Cys48

Figure S10. Crystal structure of X02A (PDB: 8BLX) showing the formation of disulphide bond between Cysteine 48 and 301

**Differential scanning fluorimetry**

Differential scanning fluorimetry (DSF) data were obtained with Sypro-Orange dye (ThermoFisher Scientific) on an Applied Biosystem 7500 Real-Time PCR system (ThermoFisher Scientific). For all enzymes a 5 µM enzyme concentration and a 5 µM dye concentration where used in 25 µl buffer solution (50 mM Tris‐HCl pH 7.4, 150 mM NaCl, 5% glycerol).

All the Tm measurements were performed at least in triplicates. Fluorescence signal was monitored during the thermal denaturation occurring to the protein upon increasing the temperature from 15°C to 95°C.

Data were normalized and fitted with the free software available at the platform <https://paulsbond.co.uk/jtsa> (1) to calculate the Tm.


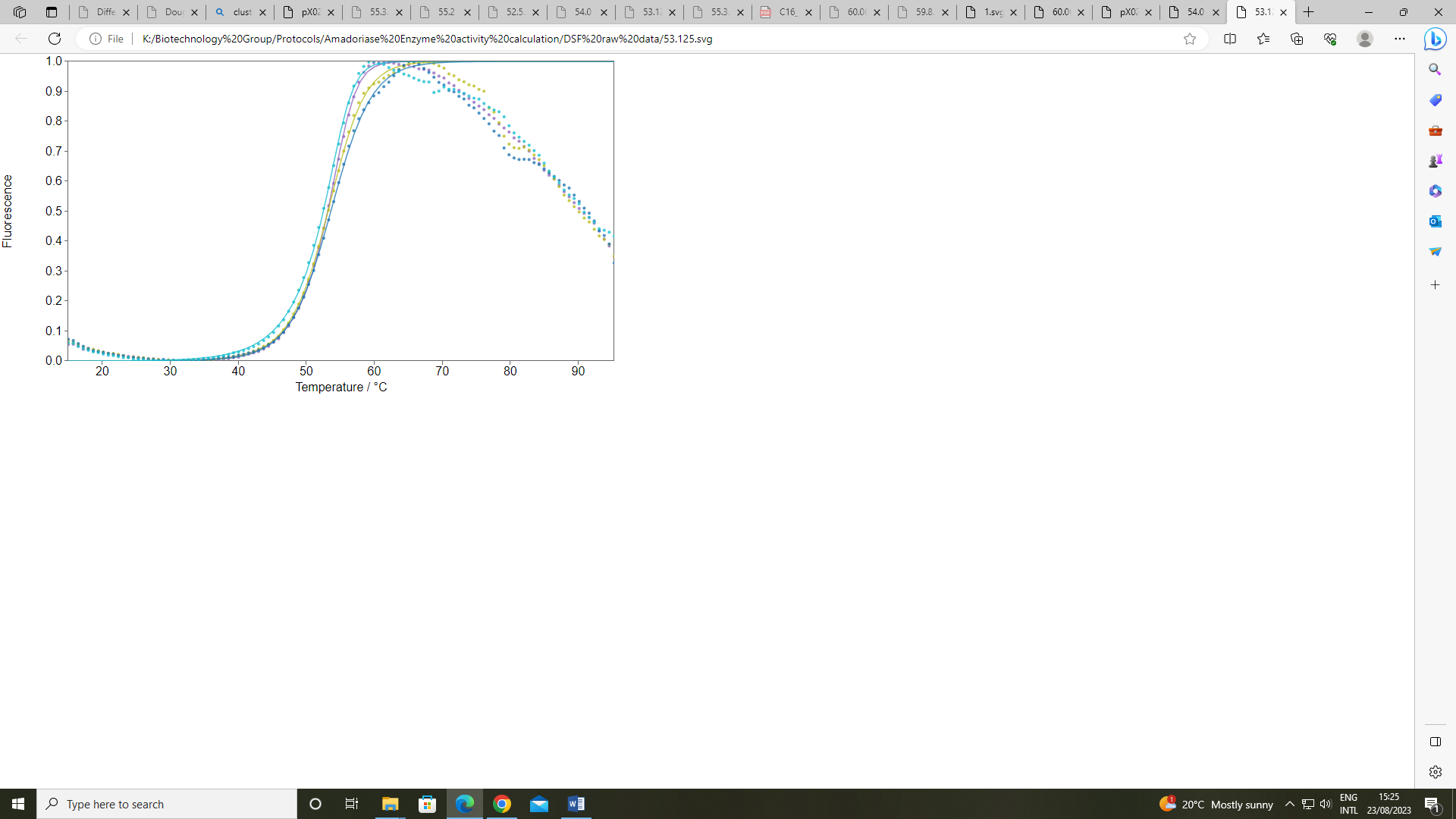


Figure S11. PnFPOX (wild type WT) Tm = 53.2 ± 0.2 °C.


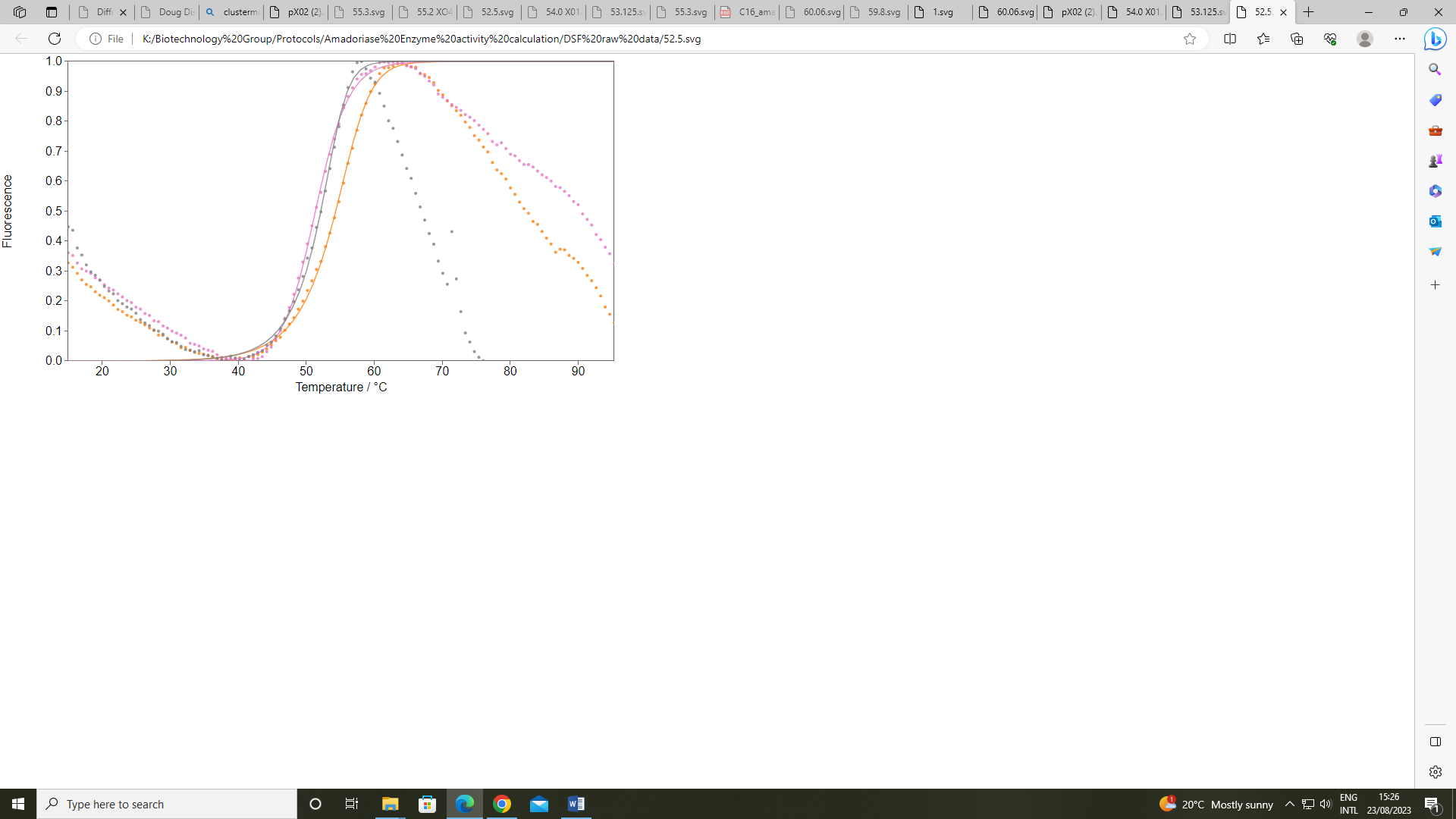


Figure S12. L3-35A Tm = 52.2 ± 0.2 °C.


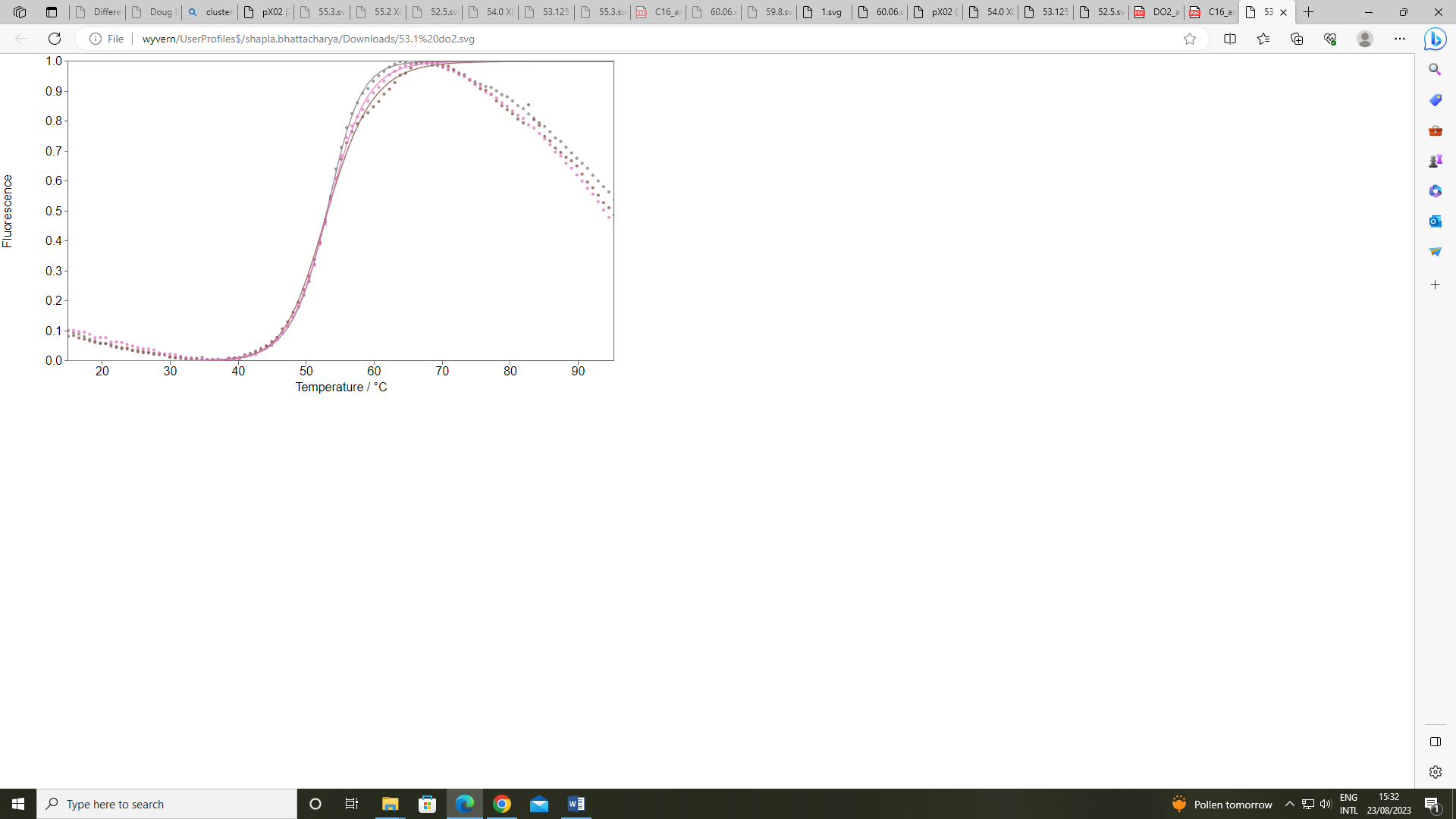


Figure S13. D02 Tm = 53.1 ± 0.5 °C.


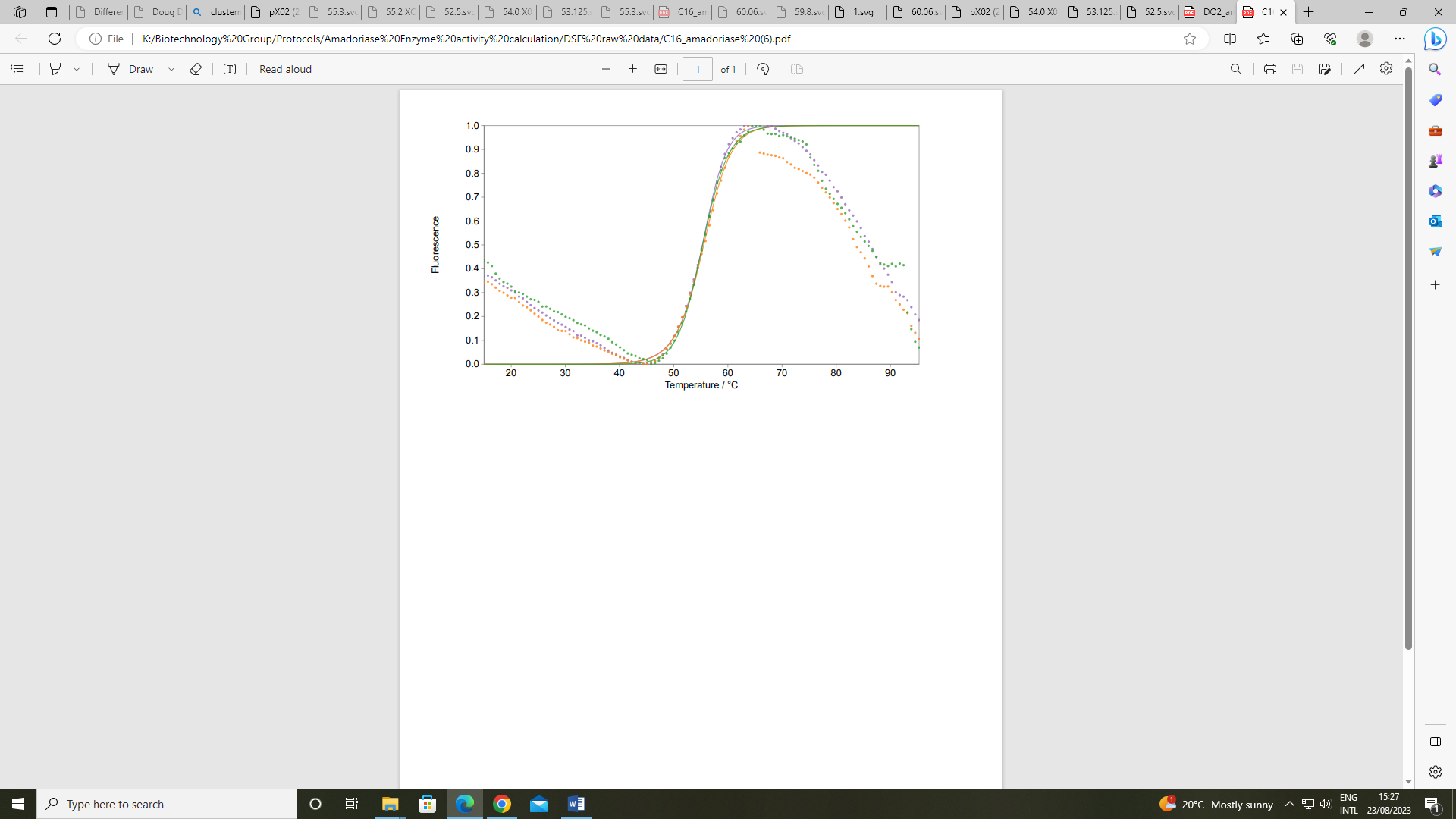


Figure S14. C16 Tm = 55.2 ± 0.1 °C.


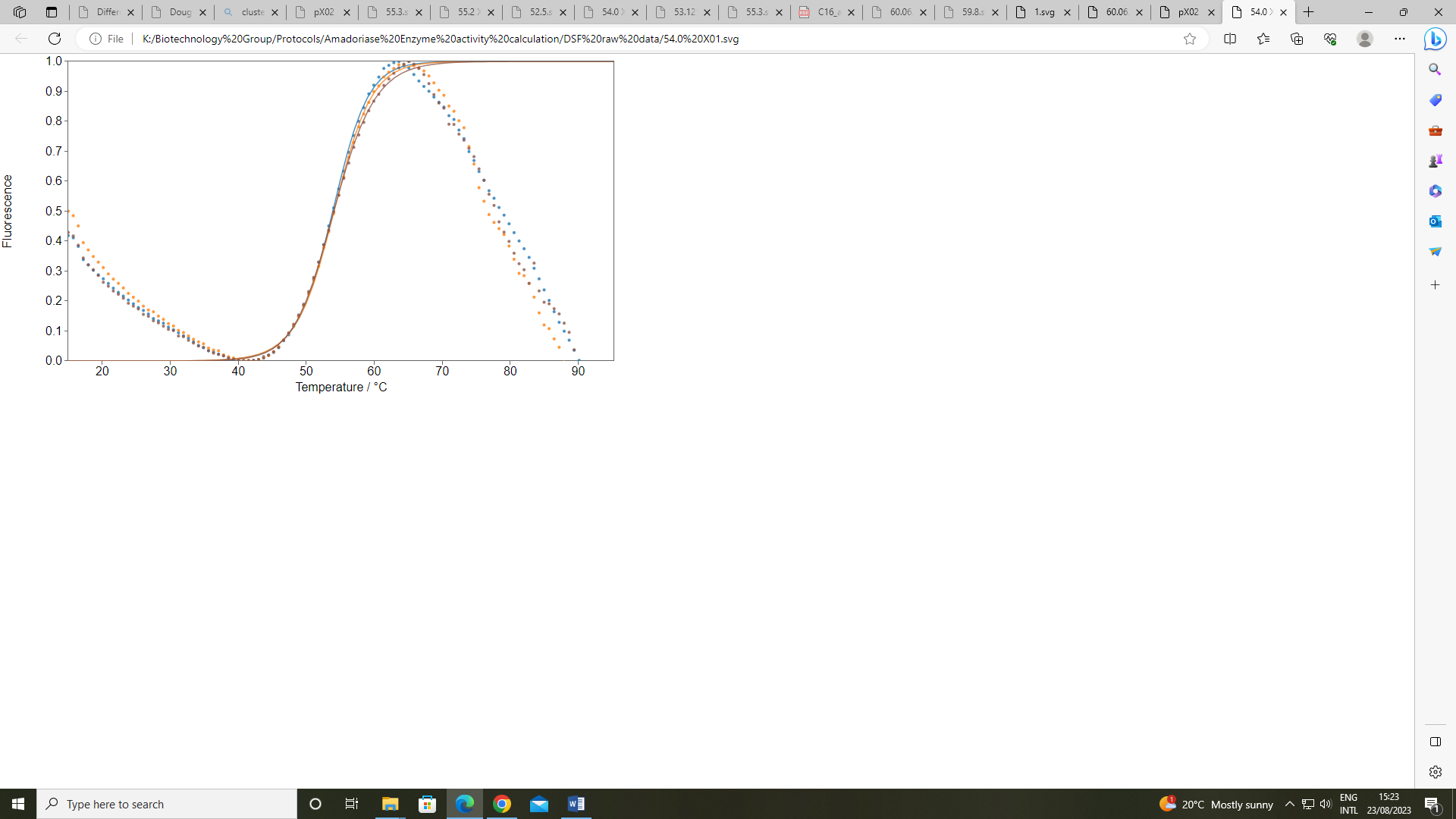


Figure S15. X01 Tm = 54.0 ± 0.1 °C.


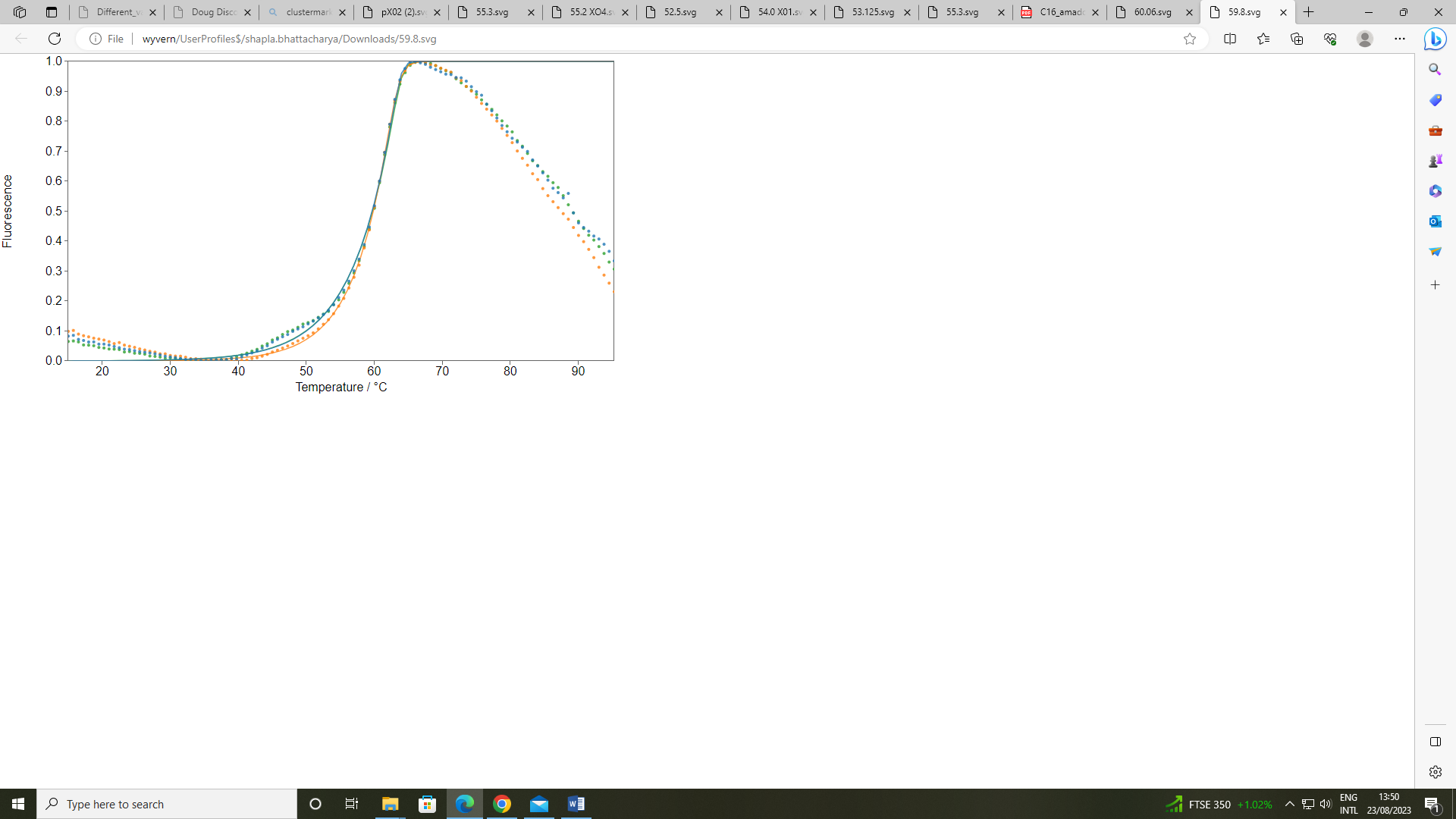


Figure S16. X02A Tm = 60.0 ± 0.3 °C.


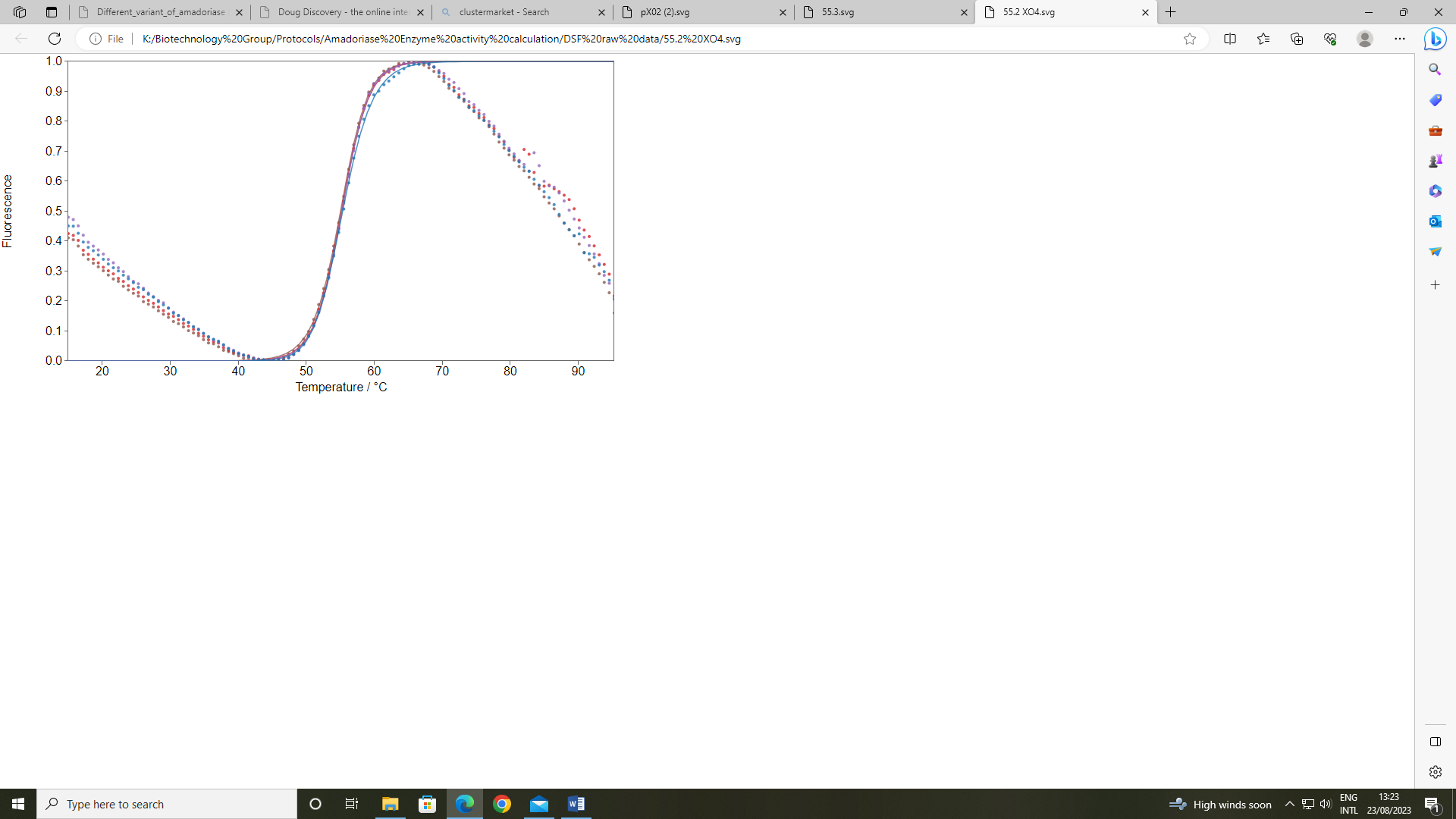


Figure S17. X04 Tm = 55.2 ± 0.3 °C.


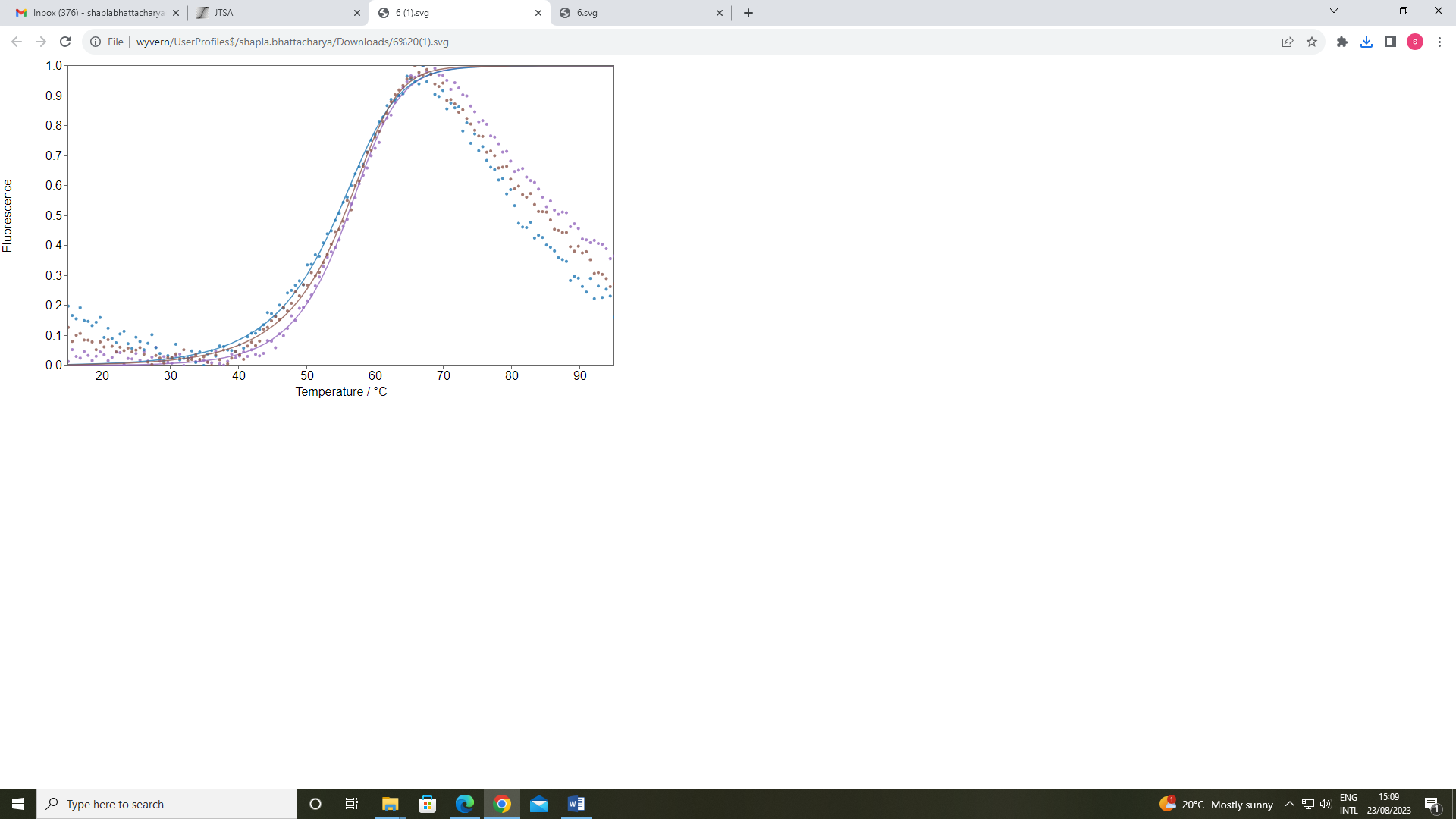


Figure S18. X07 Tm = 55.3 ± 0.4 °C.


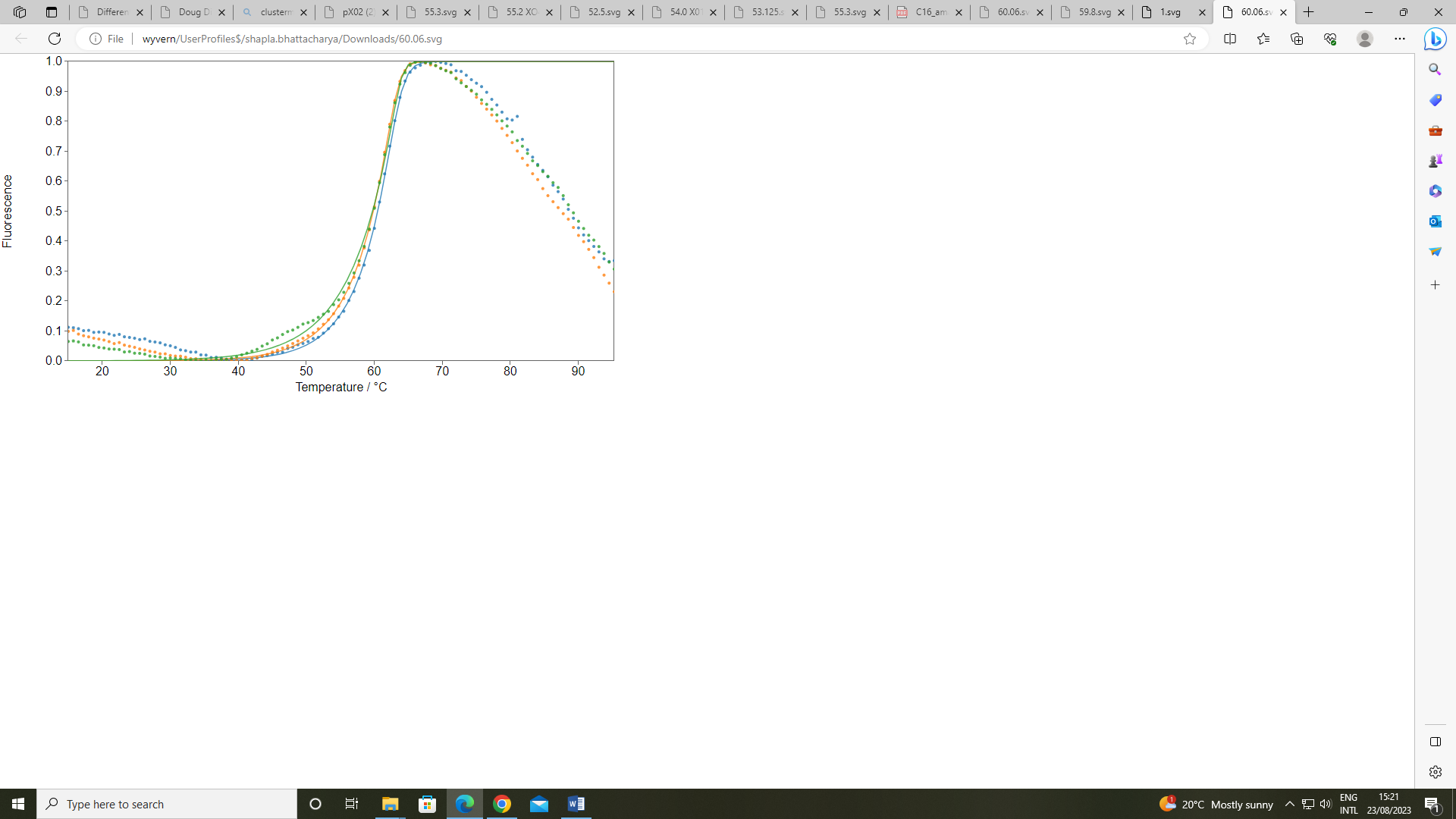


Figure S19. X02B Tm = 60.1 ± 0.6 °C.


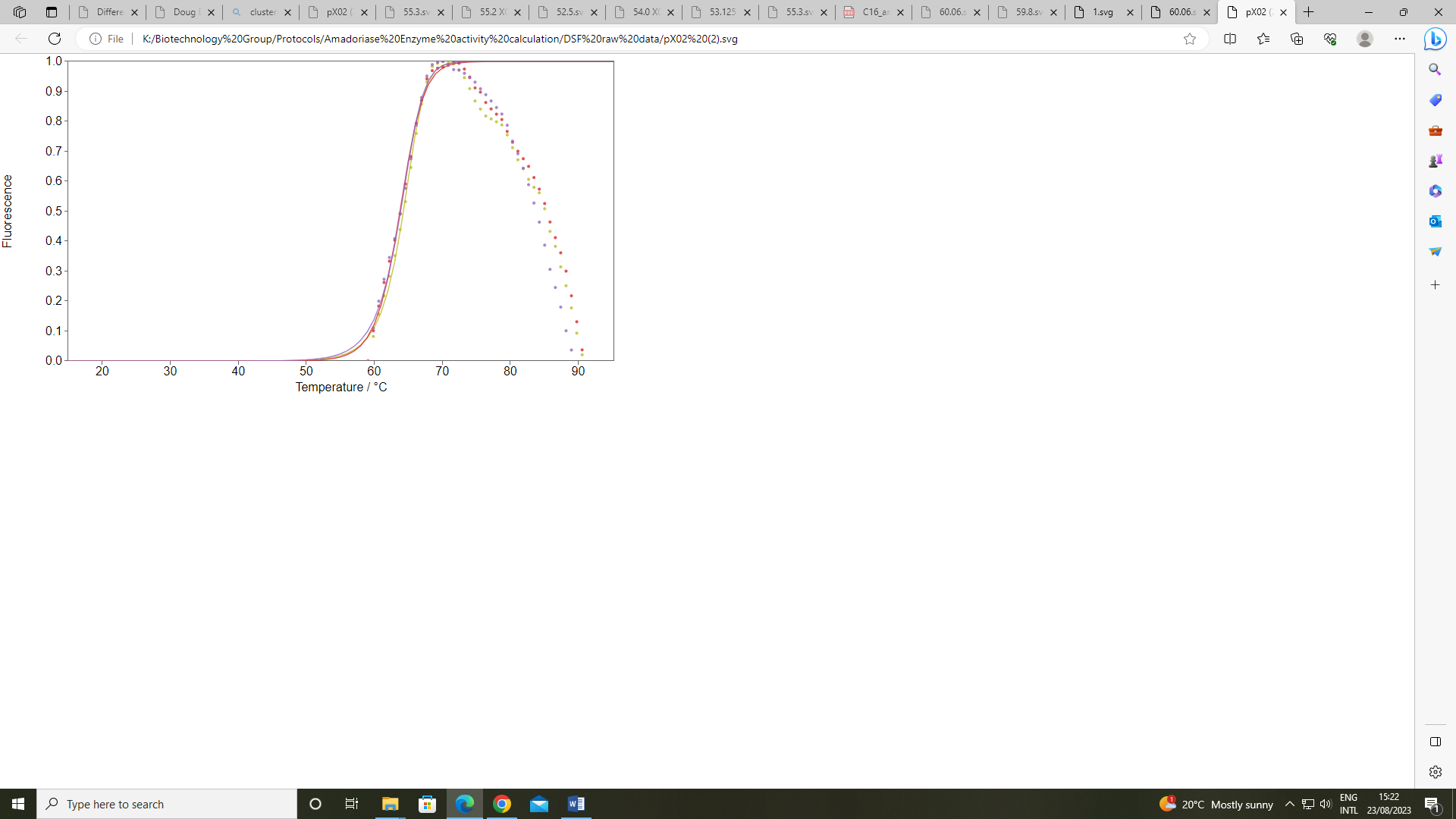


Figure S20. X02C Tm = 64.0 ± 0.2 °C.

**Table S2 – Average RMSF calculated for the engineered enzymes**

| **Design** | **Average RMSF [Å]** | **Design** | **Average RMSF [Å]** | **Design** | **Average RMSF [Å]** | **Design** | **Average RMSF [Å]** |
| --- | --- | --- | --- | --- | --- | --- | --- |
| L3-35A | 0.2235 | C01 | 0.3694 | D01 | 0.2024 | S01 | 0.2471 |
| X01 | 0.1224 | C02 | 0.2612 | D02* | 0.1200 | S02 | 0.2188 |
| X02 | 0.1435 | C03 | 0.1624 | D03 | 0.2094 | S03 | 0.1929 |
| X03 | 0.2118 | C04 | 0.1906 | D04 | 0.2329 | S04 | 0.1624 |
| X04 | 0.1294 | C05 | 0.2565 | D05 | 0.2353 | S05 | 0.2024 |
| X05 | 0.1694 | C06 | 0.2024 | D06 | 0.3459 | S06 | 0.1600 |
| X06 | 0.1694 | C07 | 0.2659 | D07 | 0.2847 | S07 | 0.2541 |
| X07 | 0.1341 | C08 | 0.2706 | D08 | 0.2776 | S08 | 0.2259 |
| X08 | 0.1788 | C09 | 0.2424 | D09 | 0.2071 | S09 | 0.1882 |
| P1 | 0.1976 | C10 | 0.2047 | D10 | 0.2871 | S10 | 0.1835 |
| P2 | 0.2188 | C11 | 0.2071 | D11 | 0.1671 | S11 | 0.1788 |
| P3 | 0.2141 | C12 | 0.2329 | D12 | 0.1718 | S12 | 0.2188 |
| P4 | 0.2541 | C13 | 0.2282 | D13 | 0.3153 | S13 | 0.2212 |
| P5 | 0.2494 | C14 | 0.3106 | D14 | 0.2259 | S14 | 0.2753 |
| P6 | 0.1906 | C15 | 0.2729 | D15 | 0.2047 | S15 | 0.2894 |
| P7 | 0.3247 | C16* | 0.1459 | D16 | 0.4612 | S16 | 0.3247 |
| P8 | 0.2306 | C17 | 0.2776 | D17 | 0.2565 | S17 | 0.2753 |
| P9 | 0.2047 | C18 | 0.1788 | D18 | 0.2988 | S18 | 0.1906 |
|  |  | C19 | 0.2518 | D19 | 0.2424 | S19 | 0.2471 |
|  |  | C20 | 0.2871 | D20 | 0.2329 | S20 | 0.2541 |

**Sequences of the designed enzymes**

>L3-35A

MAPSRANTKVIVVGGGGTIGSSTALHLVRSGYTPSNVTVLDAYPIPSSQSAGNDLNKIMDADADPAADAARQMWNEDELFKKFFHNTGRLDCAHGEKDIADLKKRYQNLVDWGLDATVEWLDSEDEILKRMPQLTRDQIKGWKAIFSKDGGWLAAAKAIKAIGEYLRDQGVRFGFYGAGSFKQPLLAEGVCIGVETVDGTRYYADKVVLAAGAWSPTLVELQEQCVSKAWVYGHIQLTPEEAARYKNSPVVYNGDVGFFFEPNEHGIIKVCDEFPGFTRFKMHQPFGAKAPKRISVPRSHAKHPTDTIPDASIVRIRRAIATFMPQFKNKPLFNQAMCWCTDTADGHLLICEHPEWKNFYLATGDSGDSFKLLPIIGKYVVELLEGTLADELAHKWRWRPGSGDALKSRREAPAKDLADMPGWNHD

>C01

MAPSRANTKVIVVGGGGTIGSSTALHLVRSGYTPSNVTVLDAYPIPSSQSAGNDLNKIMDADADPAADAARQMWKEDELFKKFFHNTGRLDCAHGEKDIADLKKRYQNLVDKGKDATVEWLDSEDEILKRMPQLTRDQIKGWKAIFSKDGGWLAAAKAIKAIGEYLRDQGVRFGFKGAGSFKQPLLAEGVCIGVETVDGTRYYADKVVLAAGAWSPTLVELQEQCVSKAWVYGHIQLTPEEAARYKNSPVVYNGDVGFFFEPNEKGIIKVCDEFPGFTRFKMHQPFGAKAPKRISVPRSHAKHPTDTIPDASIVRIRRAIATFMPQFKDKPLFNQAMCWCTDTADGHLLICEHPEWKNFYLATGDSGDSFKLLPIIGKYVVELLEGTLADELAKKWRWRPGSGDALKSRREAPAKDLADMPGWNHD

>C02

MAPSRANTKVIVVGGGGTIGSSTALHLVRSGYTPSNVTVLDAYPIPSSQSAGNDLNKIMDADADPAADAARQMWKEDELFKKFFHNTGRLDCAHGEKDIADLKKRYQNLVDKGKDATVEWLDSEDEILKRMPQLTRDQIKGWKAIFSKDGGWLAAAKAIKAIGEYLRDQGVRFGFKGAGSFKQPLLAEGVCIGVETVDGTRYYADKVVLAAGAWSPTLVELQEQCVSKAWVYGHIQLTPEEAARYKNSPVVYNGDVGFFFEPNEKGIIKVCDEFPGFTRFKMHQPFGAKAPKRISVPRSHAKHPTDTIPDASIVRIRRAIATFMPQFKDKPLFNQAMCWCTDTADGHLLICEHPEWKNFYLATGDSGDSFKLLPIIGKYVVELLEGTLADELAKKWRWRPGSGDALKSRREAPAKDLADMPGWNHD

>C03

MAPSRANTKVIVVGGGGTIGSSTALHLVRSGYTPSNVTVLDAYPIPSSQSAGNDLNKIMDADADPAADAARQMWKEDELFKKFFHNTGRLDCAHGEKDIADLKKRYQNLVDKGKDATVEWLDSEDEILKRMPQLTRDQIKGWKAIFSKDGGWLAAAKAIKAIGEYLRDQGVRFGFKGAGSFKQPLLAEGVCIGVETVDGTRYYADKVVLAAGAWSPTLVELQEQCVSKAWVYGHIQLTPEEAARYKNSPVVYNGDVGFFFEPNEKGIIKVCDEFPGFTRFKMHQPFGAKAPKRISVPRSHAKHPTDTIPDASIVRIRRAIATFMPQFKDKPLFNQAMCWCTDTADGHLLICEHPEWKNFYLATGDSGDSFKLLPIIGKYVVELLEGTLADELAKKWRWRPGSGDALKSRREAPAKDLADMPGWNHD

>C04

MAPSRANTKVIVVGGGGTIGSSTALHLVRSGYTPSNVTVLDAYPIPSSQSAGNDLNKIMDADADPAADAARQMWKEDELFKKFFHNTGRLDCAHGEKDIADLKKRYQNLVDKGKDATVEWLDSEDEILKRMPQLTRDQIKGWKAIFSKDGGWLAAAKAIKAIGEYLRDQGVRFGFKGAGSFKQPLLAEGVCIGVETVDGTRYYADKVVLAAGAWSPTLVELQEQCVSKAWVYGHIQLTPEEAARYKNSPVVYNGDVGFFFEPNEKGIIKVCDEFPGFTRFKMHQPFGAKAPKRISVPRSHAKHPTDTIPDASIVRIRRAIATFMPQFKDKPLFNQAMCWCTDTADGHLLICEHPEWKNFYLATGDSGDSFKLLPIIGKYVVELLEGTLADELAKKWRWRPGSGDALKSRREAPAKDLADMPGWNHD

>C05

MAPSRANTKVIVVGGGGTIGSSTALHLVRSGYTPSNVTVLDAYPIPSSQSAGNDLNKIMDADADPAADAARQMWKEDELFKKFFHNTGRLDCAHGEKDIADLKKRYQNLVDKGKDATVEWLDSEDEILKRMPQLTRDQIKGWKAIFSKDGGWLAAAKAIKAIGEYLRDQGVRFGFKGAGSFKQPLLAEGVCIGVETVDGTRYYADKVVLAAGAWSPTLVELQEQCVSKAWVYGHIQLTPEEAARYKNSPVVYNGDVGFFFEPNEKGIIKVCDEFPGFTRFKMHQPFGAKAPKRISVPRSHAKHPTDTIPDASIVRIRRAIATFMPQFKDKPLFNQAMCWCTDTADGHLLICEHPEWKNFYLATGDSGDSFKLLPIIGKYVVELLEGTLADELAKKWRWRPGSGDALKSRREAPAKDLADMPGWNHD

>C06

MAPSRANTKVIVVGGGGTIGSSTALHLVRSGYTPSNVTVLDAYPIPSSQSAGNDLNKIMDADADPAADAARQMWKEDELFKKFFHNTGRLDCAHGEKDIADLKKRYQNLVDKGKDATVEWLDSEDEILKRMPQLTRDQIKGWKAIFSKDGGWLAAAKAIKAIGEYLRDQGVRFGFKGAGSFKQPLLAEGVCIGVETVDGTRYYADKVVLAAGAWSPTLVELQEQCVSKAWVYGHIQLTPEEAARYKNSPVVYNGDVGFFFEPNEKGIIKVCDEFPGFTRFKMHQPFGAKAPKRISVPRSHAKHPTDTIPDASIVRIRRAIATFMPQFKDKPLFNQAMCWCTDTADGHLLICEHPEWKNFYLATGDSGDSFKLLPIIGKYVVELLEGTLADELAKKWRWRPGSGDALKSRREAPAKDLADMPGWNHD

>C07

MAPSRANTKVIVVGGGGTIGSSTALHLVRSGYTPSNVTVLDAYPIPSSQSAGNDLNKIMDADADPAADAARQMWKEDELFKKFFHNTGRLDCAHGEKDIADLKKRYQNLVDKGKDATVEWLDSEDEILKRMPQLTRDQIKGWKAIFSKDGGWLAAAKAIKAIGEYLRDQGVRFGFKGAGSFKQPLLAEGVCIGVETVDGTRYYADKVVLAAGAWSPTLVELQEQCVSKAWVYGHIQLTPEEAARYKNSPVVYNGDVGFFFEPNEKGIIKVCDEFPGFTRFKMHQPFGAKAPKRISVPRSHAKHPTDTIPDASIVRIRRAIATFMPQFKNKPLFNQAMCWCTDTADGHLLICEHPEWKNFYLATGDSGDSFKLLPIIGKYVVELLEGTLADELAKKWRWRPGSGDALKSRREAPAKDLADMPGWNHD

>C08

MAPSRANTKVIVVGGGGTIGSSTALHLVRSGYTPSNVTVLDAYPIPSSQSAGNDLNKIMDADADPAADAARQMWKEDELFKKFFHNTGRLDCAHGEKDIADLKKRYQNLVDKGKDATVEWLDSEDEILKRMPQLTRDQIKGWKAIFSKDGGWLAAAKAIKAIGEYLRDQGVRFGFKGAGSFKQPLLAEGVCIGVETVDGTRYYADKVVLAAGAWSPTLVELQEQCVSKAWVYGHIQLTPEEAARYKNSPVVYNGDVGFFFEPNEKGIIKVCDEFPGFTRFKMHQPFGAKAPKRISVPRSHAKHPTDTIPDASIVRIRRAIATFMPQFKNKPLFNQAMCWCTDTADGHLLICEHPEWKNFYLATGDSGDSFKLLPIIGKYVVELLEGTLADELAKKWRWRPGSGDALKSRREAPAKDLADMPGWNHD

>C09

MAPSRANTKVIVVGGGGTIGSSTALHLVRSGYTPSNVTVLDAYPIPSSQSAGNDLNKIMDADADPAADAARQMWKEDELFKKFFHNTGRLDCAHGEKDIADLKKRYQNLVDKGKDATVEWLDSEDEILKRMPQLTRDQIKGWKAIFSKDGGWLAAAKAIKAIGEYLRDQGVRFGFKGAGSFKQPLLAEGVCIGVETVDGTRYYADKVVLAAGAWSPTLVELQEQCVSKAWVYGHIQLTPEEAARYKNSPVVYNGDVGFFFEPNEKGIIKVCDEFPGFTRFKMHQPFGAKAPKRISVPRSHAKHPTDTIPDASIVRIRRAIATFMPQFKNKPLFNQAMCWCTDTADGHLLICEHPEWKNFYLATGDSGDSFKLLPIIGKYVVELLEGTLADELAKKWRWRPGSGDALKSRREAPAKDLADMPGWNHD

>protein.pdb

MAPSRANTKVIVVGGGGTIGSSTALHLVRSGYTPSNVTVLDAYPIPSSQSAGNDLNKIMDADADPAADAARQMWKEDELFKKFFHNTGRLDCAHGEKDIADLKKRYQNLVDKGKDATVEWLDSEDEILKRMPQLTRDQIKGWKAIFSKDGGWLAAAKAIKAIGEYLRDQGVRFGFKGAGSFKQPLLAEGVCIGVETVDGTRYYADKVVLAAGAWSPTLVELQEQCVSKAWVYGHIQLTPEEAARYKNSPVVYNGDVGFFFEPNEKGIIKVCDEFPGFTRFKMHQPFGAKAPKRISVPRSHAKHPTDTIPDASIVRIRRAIATFMPQFKDKPLFNQAMCWCTDTADGHLLICEHPEWKNFYLATGDSGDSFKLLPIIGKYVVELLEGTLADELAKKWRWRPGSGDALKSRREAPAKDLADMPGWNHD

>C10

MAPSRANTKVIVVGGGGTIGSSTALHLVRSGYTPSNVTVLDAYPIPSSQSAGNDLNKIMDADADPAADAARQMWKEDELFKKFFHNTGRLDCAHGEKDIADLKKRYQNLVDKGKDATVEWLDSEDEILKRMPQLTRDQIKGWKAIFSKDGGWLAAAKAIKAIGEYLRDQGVRFGFKGAGSFKQPLLAEGVCIGVETVDGTRYYADKVVLAAGAWSPTLVELQEQCVSKAWVYGHIQLTPEEAARYKNSPVVYNGDVGFFFEPNEKGIIKVCDEFPGFTRFKMHQPFGAKAPKRISVPRSHAKHPTDTIPDASIVRIRRAIATFMPQFKDKPLFNQAMCWCTDTADGHLLICEHPEWKNFYLATGDSGDSFKLLPIIGKYVVELLEGTLADELAKKWRWRPGSGDALKSRREAPAKDLADMPGWNHD

>C11

MAPSRANTKVIVVGGGGTIGSSTALHLVRSGYTPSNVTVLDAYPIPSSQSAGNDLNKIMDADADPAADAARQMWKEDELFKKFFHNTGRLDCAHGEKDIADLKKRYQNLVDKGKDATVEWLDSEDEILKRMPQLTRDQIKGWKAIFSKDGGWLAAAKAIKAIGEYLRDQGVRFGFKGAGSFKQPLLAEGVCIGVETVDGTRYYADKVVLAAGAWSPTLVELQEQCVSKAWVYGHIQLTPEEAARYKNSPVVYNGDVGFFFEPNEKGIIKVCDEFPGFTRFKMHQPFGAKAPKRISVPRSHAKHPTDTIPDASIVRIRRAIATFMPQFKDKPLFNQAMCWCTDTADGHLLICEHPEWKNFYLATGDSGDSFKLLPIIGKYVVELLEGTLADELAKKWRWRPGSGDALKSRREAPAKDLADMPGWNHD

>C12

MAPSRANTKVIVVGGGGTIGSSTALHLVRSGYTPSNVTVLDAYPIPSSQSAGNDLNKIMDADADPAADAARQMWKEDELFKKFFHNTGRLDCAHGEKDIADLKKRYQNLVDKGKDATVEWLDSEDEILKRMPQLTRDQIKGWKAIFSKDGGWLAAAKAIKAIGEYLRDQGVRFGFKGAGSFKQPLLAEGVCIGVETVDGTRYYADKVVLAAGAWSPTLVELQEQCVSKAWVYGHIQLTPEEAARYKNSPVVYNGDVGFFFEPNEKGIIKVCDEFPGFTRFKMHQPFGAKAPKRISVPRSHAKHPTDTIPDASIVRIRRAIATFMPQFKDKPLFNQAMCWCTDTADGHLLICEHPEWKNFYLATGDSGDSFKLLPIIGKYVVELLEGTLADELAKKWRWRPGSGDALKSRREAPAKDLADMPGWNHD

>C13

MAPSRANTKVIVVGGGGTIGSSTALHLVRSGYTPSNVTVLDAYPIPSSQSAGNDLNKIMDADADPAADAARQMWKEDELFKKFFHNTGRLDCAHGEKDIADLKKRYQNLVDKGKDATVEWLDSEDEILKRMPQLTRDQIKGWKAIFSKDGGWLAAAKAIKAIGEYLRDQGVRFGFKGAGSFKQPLLAEGVCIGVETVDGTRYYADKVVLAAGAWSPTLVELQEQCVSKAWVYGHIQLTPEEAARYKNSPVVYNGDVGFFFEPNEKGIIKVCDEFPGFTRFKMHQPFGAKAPKRISVPRSHAKHPTDTIPDASIVRIRRAIATFMPQFKDKPLFNQAMCWCTDTADGHLLICEHPEWKNFYLATGDSGDSFKLLPIIGKYVVELLEGTLADELAKKWRWRPGSGDALKSRREAPAKDLADMPGWNHD

>C14

MAPSRANTKVIVVGGGGTIGSSTALHLVRSGYTPSNVTVLDAYPIPSSQSAGNDLNKIMDADADPAADAARQMWKEDELFKKFFHNTGRLDCAHGEKDIADLKKRYQNLVDKGKDATVEWLDSEDEILKRMPQLTRDQIKGWKAIFSKDGGWLAAAKAIKAIGEYLRDQGVRFGFKGAGSFKQPLLAEGVCIGVETVDGTRYYADKVVLAAGAWSPTLVELQEQCVSKAWVYGHIQLTPEEAARYKNSPVVYNGDVGFFFEPNEKGIIKVCDEFPGFTRFKMHQPFGAKAPKRISVPRSHAKHPTDTIPDASIVRIRRAIATFMPQFKDKPLFNQAMCWCTDTADGHLLICEHPEWKNFYLATGDSGDSFKLLPIIGKYVVELLEGTLADELAKKWRWRPGSGDALKSRREAPAKDLADMPGWNHD

>C15

MAPSRANTKVIVVGGGGTIGSSTALHLVRSGYTPSNVTVLDAYPIPSSQSAGNDLNKIMDADADPAADAARQMWKEDELFKKFFHNTGRLDCAHGEKDIADLKKRYQNLVDKGKDATVEWLDSEDEILKRMPQLTRDQIKGWKAIFSKDGGWLAAAKAIKAIGEYLRDQGVRFGFKGAGSFKQPLLAEGVCIGVETVDGTRYYADKVVLAAGAWSPTLVELQEQCVSKAWVYGHIQLTPEEAARYKNSPVVYNGDVGFFFEPNEKGIIKVCDEFPGFTRFKMHQPFGAKAPKRISVPRSHAKHPTDTIPDASIVRIRRAIATFMPQFKDKPLFNQAMCWCTDTADGHLLICEHPEWKNFYLATGDSGDSFKLLPIIGKYVVELLEGTLADELAKKWRWRPGSGDALKSRREAPAKDLADMPGWNHD

>C16

MAPSRANTKVIVVGGGGTIGSSTALHLVRSGYTPSNVTVLDAYPIPSSQSAGNDLNKIMDADADPAADAARQMWKEDELFKKFFHNTGRLDCAHGEKDIADLKKRYQNLVDKGKDATVEWLDSEDEILKRMPQLTRDQIKGWKAIFSKDGGWLAAAKAIKAIGEYLRDQGVRFGFKGAGSFKQPLLAEGVCIGVETVDGTRYYADKVVLAAGAWSPTLVELQEQCVSKAWVYGHIQLTPEEAARYKNSPVVYNGDVGFFFEPNEKGIIKVCDEFPGFTRFKMHQPFGAKAPKRISVPRSHAKHPTDTIPDASIVRIRRAIATFMPQFKDKPLFNQAMCWCTDTADGHLLICEHPEWKNFYLATGDSGDSFKLLPIIGKYVVELLEGTLADELAKKWRWRPGSGDALKSRREAPAKDLADMPGWNHD

>C17

MAPSRANTKVIVVGGGGTIGSSTALHLVRSGYTPSNVTVLDAYPIPSSQSAGNDLNKIMDADADPAADAARQMWKEDELFKKFFHNTGRLDCAHGEKDIADLKKRYQNLVDKGKDATVEWLDSEDEILKRMPQLTRDQIKGWKAIFSKDGGWLAAAKAIKAIGEYLRDQGVRFGFKGAGSFKQPLLAEGVCIGVETVDGTRYYADKVVLAAGAWSPTLVELQEQCVSKAWVYGHIQLTPEEAARYKNSPVVYNGDVGFFFEPNEKGIIKVCDEFPGFTRFKMHQPFGAKAPKRISVPRSHAKHPTDTIPDASIVRIRRAIATFMPQFKDKPLFNQAMCWCTDTADGHLLICEHPEWKNFYLATGDSGDSFKLLPIIGKYVVELLEGTLADELAKKWRWRPGSGDALKSRREAPAKDLADMPGWNHD

>C18

MAPSRANTKVIVVGGGGTIGSSTALHLVRSGYTPSNVTVLDAYPIPSSQSAGNDLNKIMDADADPAADAARQMWKEDELFKKFFHNTGRLDCAHGEKDIADLKKRYQNLVDKGKDATVEWLDSEDEILKRMPQLTRDQIKGWKAIFSKDGGWLAAAKAIKAIGEYLRDQGVRFGFKGAGSFKQPLLAEGVCIGVETVDGTRYYADKVVLAAGAWSPTLVELQEQCVSKAWVYGHIQLTPEEAARYKNSPVVYNGDVGFFFEPNEKGIIKVCDEFPGFTRFKMHQPFGAKAPKRISVPRSHAKHPTDTIPDASIVRIRRAIATFMPQFKDKPLFNQAMCWCTDTADGHLLICEHPEWKNFYLATGDSGDSFKLLPIIGKYVVELLEGTLADELAKKWRWRPGSGDALKSRREAPAKDLADMPGWNHD

>C19

MAPSRANTKVIVVGGGGTIGSSTALHLVRSGYTPSNVTVLDAYPIPSSQSAGNDLNKIMDADADPAADAARQMWKEDELFKKFFHNTGRLDCAHGEKDIADLKKRYQNLVDKGKDATVEWLDSEDEILKRMPQLTRDQIKGWKAIFSKDGGWLAAAKAIKAIGEYLRDQGVRFGFKGAGSFKQPLLAEGVCIGVETVDGTRYYADKVVLAAGAWSPTLVELQEQCVSKAWVYGHIQLTPEEAARYKNSPVVYNGDVGFFFEPNEKGIIKVCDEFPGFTRFKMHQPFGAKAPKRISVPRSHAKHPTDTIPDASIVRIRRAIATFMPQFKDKPLFNQAMCWCTDTADGHLLICEHPEWKNFYLATGDSGDSFKLLPIIGKYVVELLEGTLADELAKKWRWRPGSGDALKSRREAPAKDLADMPGWNHD

>C20

MAPSRANTKVIVVGGGGTIGSSTALHLVRSGYTPSNVTVLDAYPIPSSQSAGNDLNKIMDADADPAADAARQMWKEDELFKKFFHNTGRLDCAHGEKDIADLKKRYQNLVDKGKDATVEWLDSEDEILKRMPQLTRDQIKGWKAIFSKDGGWLAAAKAIKAIGEYLRDQGVRFGFKGAGSFKQPLLAEGVCIGVETVDGTRYYADKVVLAAGAWSPTLVELQEQCVSKAWVYGHIQLTPEEAARYKNSPVVYNGDVGFFFEPNEKGIIKVCDEFPGFTRFKMHQPFGAKAPKRISVPRSHAKHPTDTIPDASIVRIRRAIATFMPQFKDKPLFNQAMCWCTDTADGHLLICEHPEWKNFYLATGDSGDSFKLLPIIGKYVVELLEGTLADELAKKWRWRPGSGDALKSRREAPAKDLADMPGWNHD

>D01

MAPSRANTKVIVVGGGGTIGSSTALHLVRSGYTPSNVTVLDAYPIPSSQSAGNDLNKIMDADADPAADAARQMWNEDELFKKFFHNTGRLDCAHGEKDIADLKKRYQNLVSGGLDATVEWLDSEDEILKRMPQLTRDQIKGWKAIFSKDGGWLAAAKAIKAIGEYLRDQGVRFGFYGAGSFKQPLLAEGVCIGVETVDGTRYYADKVVLAAGAWSPTLVELQEQCVSKAWVYGHIQLTPEEAARYKNSPVVYNGDVGFFFEPNEHGIIKVCDEFPGFTRFKMHQPFGAKAPKRISVPRSHAKHPTDTIPDASIVRIRRAIATFMPQFKNKPLFNQAMCWCTDTADGHLLICEHPEWKNFYLATGDSGDSFKLLPIIGKYVVELLEGTLADELAHKWRWRPGSGDALKSRREAPAKDLADMPGWNHD

>D02

MAPSRANTKVIVVGGGGTIGSSTALHLVRSGYTPSNVTVLDAYPIPSSQSAGNDLNKIMDADADPAADAARQMWNEDELFKKFFHNTGRLDCAHGEKDIADLKKRYQNLRDWGLGATVEWLDSEDEILKRMPQLTRDQIKGWKAIFSKDGGWLAAAKAIKAIGEYLRDQGVRFGFYGAGSFKQPLLAEGVCIGVETVDGTRYYADKVVLAAGAWSPTLVELQEQCVSKAWVYGHIQLTPEEAARYKNSPVVYNGDVGFFFEPNEHGIIKVCDEFPGFTRFKMHQPFGAKAPKRISVPRSHAKHPTDTIPDASIVRIRRAIATFMPQFKNKPLFNQAMCWCTDTADGHLLICEHPEWKNFYLATGDSGDSFKLLPIIGKYVVELLEGTLADELAHKWRWRPGSGDALKSRREAPAKDLADMPGWNHD

>D03

MAPSRANTKVIVVGGGGTIGSSTALHLVRSGYTPSNVTVLDAYPIPSSQSAGNDLNKIMDADADPAADAARQMWNEDELFKKFFHNTGRLDCAHGEKDIADLKKRYQNLVWGGLDATVEWLDSEDEILKRMPQLTRDQIKGWKAIFSKDGGWLAAAKAIKAIGEYLRDQGVRFGFYGAGSFKQPLLAEGVCIGVETVDGTRYYADKVVLAAGAWSPTLVELQEQCVSKAWVYGHIQLTPEEAARYKNSPVVYNGDVGFFFEPNEHGIIKVCDEFPGFTRFKMHQPFGAKAPKRISVPRSHAKHPTDTIPDASIVRIRRAIATFMPQFKNKPLFNQAMCWCTDTADGHLLICEHPEWKNFYLATGDSGDSFKLLPIIGKYVVELLEGTLADELAHKWRWRPGSGDALKSRREAPAKDLADMPGWNHD

>D04

MAPSRANTKVIVVGGGGTIGSSTALHLVRSGYTPSNVTVLDAYPIPSSQSAGNDLNKIMDADADPAADAARQMWNEDELFKKFFHNTGRLDCAHGEKDIADLKKRYQNLVDWGLSATHEWLDSEDEILKRMPQLTRDQIKGWKAIFSKDGGWLAAAKAIKAIGEYLRDQGVRFGFYGAGSFKQPLLAEGVCIGVETVDGTRYYADKVVLAAGAWSPTLVELQEQCVSKAWVYGHIQLTPEEAARYKNSPVVYNGDVGFFFEPNEHGIIKVCDEFPGFTRFKMHQPFGAKAPKRISVPRSHAKHPTDTIPDASIVRIRRAIATFMPQFKNKPLFNQAMCWCTDTADGHLLICEHPEWKNFYLATGDSGDSFKLLPIIGKYVVELLEGTLADELAHKWRWRPGSGDALKSRREAPAKDLADMPGWNHD

>D05

MAPSRANTKVIVVGGGGTIGSSTALHLVRSGYTPSNVTVLDAYPIPSSQSAGNDLNKIMDADADPAADAARQMWNEDELFKKFFHNTGRLDCAHGEKDIADLKKRYQNLVRGGLDATVEWLDSEDEILKRMPQLTRDQIKGWKAIFSKDGGWLAAAKAIKAIGEYLRDQGVRFGFYGAGSFKQPLLAEGVCIGVETVDGTRYYADKVVLAAGAWSPTLVELQEQCVSKAWVYGHIQLTPEEAARYKNSPVVYNGDVGFFFEPNEHGIIKVCDEFPGFTRFKMHQPFGAKAPKRISVPRSHAKHPTDTIPDASIVRIRRAIATFMPQFKNKPLFNQAMCWCTDTADGHLLICEHPEWKNFYLATGDSGDSFKLLPIIGKYVVELLEGTLADELAHKWRWRPGSGDALKSRREAPAKDLADMPGWNHD

>D06

MAPSRANTKVIVVGGGGTIGSSTALHLVRSGYTPSNVTVLDAYPIPSSQSAGNDLNKIMDADADPAADAARQMWNEDELFKKFFHNTGRLDCAHGEKDIADLKKRYQNLVDWGGDGTVEWLDSEDEILKRMPQLTRDQIKGWKAIFSKDGGWLAAAKAIKAIGEYLRDQGVRFGFYGAGSFKQPLLAEGVCIGVETVDGTRYYADKVVLAAGAWSPTLVELQEQCVSKAWVYGHIQLTPEEAARYKNSPVVYNGDVGFFFEPNEHGIIKVCDEFPGFTRFKMHQPFGAKAPKRISVPRSHAKHPTDTIPDASIVRIRRAIATFMPQFKNKPLFNQAMCWCTDTADGHLLICEHPEWKNFYLATGDSGDSFKLLPIIGKYVVELLEGTLADELAHKWRWRPGSGDALKSRREAPAKDLADMPGWNHD

>D07

MAPSRANTKVIVVGGGGTIGSSTALHLVRSGYTPSNVTVLDAYPIPSSQSAGNDLNKIMDADADPAADAARQMWNEDELFKKFFHNTGRLDCAHGEKDIADLKKRYQNLVKGGLDATVEWLDSEDEILKRMPQLTRDQIKGWKAIFSKDGGWLAAAKAIKAIGEYLRDQGVRFGFYGAGSFKQPLLAEGVCIGVETVDGTRYYADKVVLAAGAWSPTLVELQEQCVSKAWVYGHIQLTPEEAARYKNSPVVYNGDVGFFFEPNEHGIIKVCDEFPGFTRFKMHQPFGAKAPKRISVPRSHAKHPTDTIPDASIVRIRRAIATFMPQFKNKPLFNQAMCWCTDTADGHLLICEHPEWKNFYLATGDSGDSFKLLPIIGKYVVELLEGTLADELAHKWRWRPGSGDALKSRREAPAKDLADMPGWNHD

>D08

MAPSRANTKVIVVGGGGTIGSSTALHLVRSGYTPSNVTVLDAYPIPSSQSAGNDLNKIMDADADPAADAARQMWNEDELFKKFFHNTGRLDCAHGEKDIADLKKRYQNLVTGGLDATVEWLDSEDEILKRMPQLTRDQIKGWKAIFSKDGGWLAAAKAIKAIGEYLRDQGVRFGFYGAGSFKQPLLAEGVCIGVETVDGTRYYADKVVLAAGAWSPTLVELQEQCVSKAWVYGHIQLTPEEAARYKNSPVVYNGDVGFFFEPNEHGIIKVCDEFPGFTRFKMHQPFGAKAPKRISVPRSHAKHPTDTIPDASIVRIRRAIATFMPQFKNKPLFNQAMCWCTDTADGHLLICEHPEWKNFYLATGDSGDSFKLLPIIGKYVVELLEGTLADELAHKWRWRPGSGDALKSRREAPAKDLADMPGWNHD

>D09

MAPSRANTKVIVVGGGGTIGSSTALHLVRSGYTPSNVTVLDAYPIPSSQSAGNDLNKIMDADADPAADAARQMWNEDELFKKFFHNTGRLDCAHGEKDIADLKKRYQNLVDWGGDTTVEWLDSEDEILKRMPQLTRDQIKGWKAIFSKDGGWLAAAKAIKAIGEYLRDQGVRFGFYGAGSFKQPLLAEGVCIGVETVDGTRYYADKVVLAAGAWSPTLVELQEQCVSKAWVYGHIQLTPEEAARYKNSPVVYNGDVGFFFEPNEHGIIKVCDEFPGFTRFKMHQPFGAKAPKRISVPRSHAKHPTDTIPDASIVRIRRAIATFMPQFKNKPLFNQAMCWCTDTADGHLLICEHPEWKNFYLATGDSGDSFKLLPIIGKYVVELLEGTLADELAHKWRWRPGSGDALKSRREAPAKDLADMPGWNHD

>D10

MAPSRANTKVIVVGGGGTIGSSTALHLVRSGYTPSNVTVLDAYPIPSSQSAGNDLNKIMDADADPAADAARQMWNEDELFKKFFHNTGRLDCAHGEKDIADLKKRYQNLVDWGGDKTVEWLDSEDEILKRMPQLTRDQIKGWKAIFSKDGGWLAAAKAIKAIGEYLRDQGVRFGFYGAGSFKQPLLAEGVCIGVETVDGTRYYADKVVLAAGAWSPTLVELQEQCVSKAWVYGHIQLTPEEAARYKNSPVVYNGDVGFFFEPNEHGIIKVCDEFPGFTRFKMHQPFGAKAPKRISVPRSHAKHPTDTIPDASIVRIRRAIATFMPQFKNKPLFNQAMCWCTDTADGHLLICEHPEWKNFYLATGDSGDSFKLLPIIGKYVVELLEGTLADELAHKWRWRPGSGDALKSRREAPAKDLADMPGWNHD

>D11

MAPSRANTKVIVVGGGGTIGSSTALHLVRSGYTPSNVTVLDAYPIPSSQSAGNDLNKIMDADADPAADAARQMWNEDELFKKFFHNTGRLDCAHGEKDIADLKKRYQNLVAGGLDATVEWLDSEDEILKRMPQLTRDQIKGWKAIFSKDGGWLAAAKAIKAIGEYLRDQGVRFGFYGAGSFKQPLLAEGVCIGVETVDGTRYYADKVVLAAGAWSPTLVELQEQCVSKAWVYGHIQLTPEEAARYKNSPVVYNGDVGFFFEPNEHGIIKVCDEFPGFTRFKMHQPFGAKAPKRISVPRSHAKHPTDTIPDASIVRIRRAIATFMPQFKNKPLFNQAMCWCTDTADGHLLICEHPEWKNFYLATGDSGDSFKLLPIIGKYVVELLEGTLADELAHKWRWRPGSGDALKSRREAPAKDLADMPGWNHD

>D12

MAPSRANTKVIVVGGGGTIGSSTALHLVRSGYTPSNVTVLDAYPIPSSQSAGNDLNKIMDADADPAADAARQMWNEDELFKKFFHNTGRLDCAHGEKDIADLKKRYQNLVDWGGDNTVEWLDSEDEILKRMPQLTRDQIKGWKAIFSKDGGWLAAAKAIKAIGEYLRDQGVRFGFYGAGSFKQPLLAEGVCIGVETVDGTRYYADKVVLAAGAWSPTLVELQEQCVSKAWVYGHIQLTPEEAARYKNSPVVYNGDVGFFFEPNEHGIIKVCDEFPGFTRFKMHQPFGAKAPKRISVPRSHAKHPTDTIPDASIVRIRRAIATFMPQFKNKPLFNQAMCWCTDTADGHLLICEHPEWKNFYLATGDSGDSFKLLPIIGKYVVELLEGTLADELAHKWRWRPGSGDALKSRREAPAKDLADMPGWNHD

>D13

MAPSRANTKVIVVGGGGTIGSSTALHLVRSGYTPSNVTVLDAYPIPSSQSAGNDLNKIMDADADPAADAARQMWNEDELFKKFFHNTGRLDCAHGEKDIADLKKRYQNLVYWGLDATGEWLDSEDEILKRMPQLTRDQIKGWKAIFSKDGGWLAAAKAIKAIGEYLRDQGVRFGFYGAGSFKQPLLAEGVCIGVETVDGTRYYADKVVLAAGAWSPTLVELQEQCVSKAWVYGHIQLTPEEAARYKNSPVVYNGDVGFFFEPNEHGIIKVCDEFPGFTRFKMHQPFGAKAPKRISVPRSHAKHPTDTIPDASIVRIRRAIATFMPQFKNKPLFNQAMCWCTDTADGHLLICEHPEWKNFYLATGDSGDSFKLLPIIGKYVVELLEGTLADELAHKWRWRPGSGDALKSRREAPAKDLADMPGWNHD

>D14

MAPSRANTKVIVVGGGGTIGSSTALHLVRSGYTPSNVTVLDAYPIPSSQSAGNDLNKIMDADADPAADAARQMWNEDELFKKFFHNTGRLDCAHGEKDIADLKKRYQNLVDWGGDETVEWLDSEDEILKRMPQLTRDQIKGWKAIFSKDGGWLAAAKAIKAIGEYLRDQGVRFGFYGAGSFKQPLLAEGVCIGVETVDGTRYYADKVVLAAGAWSPTLVELQEQCVSKAWVYGHIQLTPEEAARYKNSPVVYNGDVGFFFEPNEHGIIKVCDEFPGFTRFKMHQPFGAKAPKRISVPRSHAKHPTDTIPDASIVRIRRAIATFMPQFKNKPLFNQAMCWCTDTADGHLLICEHPEWKNFYLATGDSGDSFKLLPIIGKYVVELLEGTLADELAHKWRWRPGSGDALKSRREAPAKDLADMPGWNHD

>D15

MAPSRANTKVIVVGGGGTIGSSTALHLVRSGYTPSNVTVLDAYPIPSSQSAGNDLNKIMDADADPAADAARQMWNEDELFKKFFHNTGRLDCAHGEKDIADLKKRYQNLVDWGLSATAEWLDSEDEILKRMPQLTRDQIKGWKAIFSKDGGWLAAAKAIKAIGEYLRDQGVRFGFYGAGSFKQPLLAEGVCIGVETVDGTRYYADKVVLAAGAWSPTLVELQEQCVSKAWVYGHIQLTPEEAARYKNSPVVYNGDVGFFFEPNEHGIIKVCDEFPGFTRFKMHQPFGAKAPKRISVPRSHAKHPTDTIPDASIVRIRRAIATFMPQFKNKPLFNQAMCWCTDTADGHLLICEHPEWKNFYLATGDSGDSFKLLPIIGKYVVELLEGTLADELAHKWRWRPGSGDALKSRREAPAKDLADMPGWNHD

>D16

MAPSRANTKVIVVGGGGTIGSSTALHLVRSGYTPSNVTVLDAYPIPSSQSAGNDLNKIMDADADPAADAARQMWNEDELFKKFFHNTGRLDCAHGEKDIADLKKRYQNLRDWGLSATVEWLDSEDEILKRMPQLTRDQIKGWKAIFSKDGGWLAAAKAIKAIGEYLRDQGVRFGFYGAGSFKQPLLAEGVCIGVETVDGTRYYADKVVLAAGAWSPTLVELQEQCVSKAWVYGHIQLTPEEAARYKNSPVVYNGDVGFFFEPNEHGIIKVCDEFPGFTRFKMHQPFGAKAPKRISVPRSHAKHPTDTIPDASIVRIRRAIATFMPQFKNKPLFNQAMCWCTDTADGHLLICEHPEWKNFYLATGDSGDSFKLLPIIGKYVVELLEGTLADELAHKWRWRPGSGDALKSRREAPAKDLADMPGWNHD

>D17

MAPSRANTKVIVVGGGGTIGSSTALHLVRSGYTPSNVTVLDAYPIPSSQSAGNDLNKIMDADADPAADAARQMWNEDELFKKFFHNTGRLDCAHGEKDIADLKKRYQNLVDWGLSATTEWLDSEDEILKRMPQLTRDQIKGWKAIFSKDGGWLAAAKAIKAIGEYLRDQGVRFGFYGAGSFKQPLLAEGVCIGVETVDGTRYYADKVVLAAGAWSPTLVELQEQCVSKAWVYGHIQLTPEEAARYKNSPVVYNGDVGFFFEPNEHGIIKVCDEFPGFTRFKMHQPFGAKAPKRISVPRSHAKHPTDTIPDASIVRIRRAIATFMPQFKNKPLFNQAMCWCTDTADGHLLICEHPEWKNFYLATGDSGDSFKLLPIIGKYVVELLEGTLADELAHKWRWRPGSGDALKSRREAPAKDLADMPGWNHD

>D18

MAPSRANTKVIVVGGGGTIGSSTALHLVRSGYTPSNVTVLDAYPIPSSQSAGNDLNKIMDADADPAADAARQMWNEDELFKKFFHNTGRLDCAHGEKDIADLKKRYQNLVNGGLDATVEWLDSEDEILKRMPQLTRDQIKGWKAIFSKDGGWLAAAKAIKAIGEYLRDQGVRFGFYGAGSFKQPLLAEGVCIGVETVDGTRYYADKVVLAAGAWSPTLVELQEQCVSKAWVYGHIQLTPEEAARYKNSPVVYNGDVGFFFEPNEHGIIKVCDEFPGFTRFKMHQPFGAKAPKRISVPRSHAKHPTDTIPDASIVRIRRAIATFMPQFKNKPLFNQAMCWCTDTADGHLLICEHPEWKNFYLATGDSGDSFKLLPIIGKYVVELLEGTLADELAHKWRWRPGSGDALKSRREAPAKDLADMPGWNHD

>D19

MAPSRANTKVIVVGGGGTIGSSTALHLVRSGYTPSNVTVLDAYPIPSSQSAGNDLNKIMDADADPAADAARQMWNEDELFKKFFHNTGRLDCAHGEKDIADLKKRYQNLVDWGLSATNEWLDSEDEILKRMPQLTRDQIKGWKAIFSKDGGWLAAAKAIKAIGEYLRDQGVRFGFYGAGSFKQPLLAEGVCIGVETVDGTRYYADKVVLAAGAWSPTLVELQEQCVSKAWVYGHIQLTPEEAARYKNSPVVYNGDVGFFFEPNEHGIIKVCDEFPGFTRFKMHQPFGAKAPKRISVPRSHAKHPTDTIPDASIVRIRRAIATFMPQFKNKPLFNQAMCWCTDTADGHLLICEHPEWKNFYLATGDSGDSFKLLPIIGKYVVELLEGTLADELAHKWRWRPGSGDALKSRREAPAKDLADMPGWNHD

>D20

MAPSRANTKVIVVGGGGTIGSSTALHLVRSGYTPSNVTVLDAYPIPSSQSAGNDLNKIMDADADPAADAARQMWNEDELFKKFFHNTGRLDCAHGEKDIADLKKRYQNLVDWGLGATIEWLDSEDEILKRMPQLTRDQIKGWKAIFSKDGGWLAAAKAIKAIGEYLRDQGVRFGFYGAGSFKQPLLAEGVCIGVETVDGTRYYADKVVLAAGAWSPTLVELQEQCVSKAWVYGHIQLTPEEAARYKNSPVVYNGDVGFFFEPNEHGIIKVCDEFPGFTRFKMHQPFGAKAPKRISVPRSHAKHPTDTIPDASIVRIRRAIATFMPQFKNKPLFNQAMCWCTDTADGHLLICEHPEWKNFYLATGDSGDSFKLLPIIGKYVVELLEGTLADELAHKWRWRPGSGDALKSRREAPAKDLADMPGWNHD

>P1

MAPSRANTKVIVVGGGGTIGSSTALHLVRSGYTPSNVTVLDAYPIPSSQSAGNDLNKIMDADADPAADAARQMWNEDPLFKKFFHNTGRLDCAHGEKGIADLKKRYQNLVDAGLGATVEWLDSEDEILKRMPQLTRDQIKGWKAIFSKDGGWLAAAKAIKAIGEYLRDQGVRFGFGGAGSFKQPLLAEGVCIGVETVDGTRYYADKVVLAAGAWSPTLVELQGQCVSKAWVYGHIQLTPEEAARYKNSPVVYNGDVGFFFEPNEHGIIKVCDEFPGFTNFKMHQPFGAKAPKRISVPRSHAKHPTDTIPDASIVRIRRAIATFMPQFKNKPLFNQAMCWCTDTADGHLLICEHPEWKNFYLATGDSGHSFKLLPIIGKYVVELLEGTLADELAHKWRWRPGSGDALKSRREAPAKDLADMPGWNHD

>P2

MAPSRANTKVIVVGGGGTIGSSTALHLVRSGYTPSNVTVLDAYPIPSSQSAGNDLNKIMDADADPAADAARQMWNEDPLFKKFFHNTGRLDCAHGEKGIADLKKRYQNLVDAGLGGTVEWLDSEDEILKRMPQLTRDQIKGWKAIFSKDGGWLAAAKAIKAIGEYLRDQGVRFGFGGAGSFKQPLLANGVCIGVETVDGTRYYADKVVLAAGAWSPTLVELQGQCVSKAWVYGHIQLTPEEAARYKNSPVVYNGDVGFFFEPNEHGIIKVCDEFPGFTNFKMHQPFGAKAPKRISVPRSHAKHPTDTIPDASEERIRRAIATFMPQFKNKPLFNQAMCWCTDTADGHLLICEHPEWKNFYLATGDSGHSFKLLPIIGKYVVELLEGTLADELAHKWRWRPGSGDALKSRREAPAKDLADMPGWNHD

>P3

MAPSRANTKVIVVGGGGTIGSSTALHLVRSGYTPSNITVLDAYPIPSSQSAGNDLNKIMDADADPAADAARQMWNEDPLFKKFFHNTGRLDCAHGEKGIADLKKRYQNLVDAGLGGTVEWLDSEDEILKRMPQLTRDQIKGWKAIFSKDGGWLAAAKAIKAIGEYLRDQGVRFGFGGAGSFKQPLLANGVCIGVETVDGTRYYADKVVLAAGAWSPTLVELQGQCVSKAWVYGHIQLTPEEAARYKNSPVVYNGDVGFFFEPNEHGIIKVCDEFPGFTNFKMHQPFGAPAPKRISVPRSHAKHPTDTIPDASEERIRRAIATFMPQFKNKPLFNQAMCWCTDTADGHLLICEHPEWKNFYLATGDSGHSFKLLPIIGKYVVELLEGTLADELAHKWRWRPGSGDALKSRREAPAKDLADMPGWNHD

>P4

MAPSRANTKVIVVGGGGTIGSSTALHLVRSGYTPSNITVLDAYPIPSSQSAGNDLNKIMDADADPAADAARQMWKEDPLFKKFFHNTGRLDCAHGEKGIADLKKRYQNLVDAGLGGTVEWLDSEDEILKRMPQLTRDQIKGWKAIFSKDGGWLAAAKAIKAIGEYLRDQGVRFGFGGAGSFKQPLLENGVCIGVETVDGTRYYADKVVLAAGAWSPTLVELQGQCVSKAWVYGHIQLTPEEAARYKNSPVVYNGDVGFFFEPNEHGIIKVCDEFPGFTNFKMHQPFGAPHPKRISVPRSHAKHPTDTIPDAAEERIRRAIATFMPQFKNKPLFNQAMCWCTDTPDGHLLICEHPEWKNFYLATGDSGHSFKLLPIIGKYVVELLEGTLADELAHKWRWRPGSGDALKSRREAPAKDLADMPGWNHD

>P5

MAPSRANTKVIVVGGGGTIGSSTALHLVRSGYTPSNITVLDAYPIPSSQSAGNDLNKIMDADADPAADAARQMWKEDPLFKKFFHNTGRLDCAHGEKGIADLKKRYQNLVDAGLGGTVEWLDSEDEILKRMPQLTRDQIKGWKAIFSKDGGWLAAAKAIKAIGEYLRDQGVRFGFGGAGSFKQPLLENGVCIGVETVDGTRYYADKVVLAAGAWSPTLVELQGQCVSKAWVYGHIQLTPEEAARYKNSPVVYNGDVGFFFEPNEHGIIKVCDEFPGFTNFKMHQPFGAPHPKRISVPRSHAKHPTDTIPDAAEERIRRAIATFMPQFKNKPLFNQAMCWCTDTPDGHLLICEHPEWKNFYLATGDSGHSFKLLPIIGKYVVELLEGTLADELAHKWRWRPGSGDALKSRREAPAKDLADMPGWNHD

>P6

MAPSRKNTKVIVVGGGGTIGSSTALHLVRSGYTPSNITVLDAYPIPSSQSAGNDLNKIMDADADPAADAARQMWKNDPLFKPFFHNTGRLDCAHGEKGIAELKKRYQNLVDAGLGGNVEWLDSEDEILKRMPQLTRDQIKGWKAIFSKDGGWLAAAKAIKAIGEYLRDQGVRFGFGGAGSFKQPLLENGVCIGVETVDGTRYYADKVVLAAGAWSPTLVELQGQCVSKAWVYGHIQLTPEEAARYKNCPVVYNGDVGFFFEPNEHGIIKVCDEFPGFTNFKMHQPFGAPHPKRISVPRSHAKHPTDTIPDEAEERIRRAIRTFMPQFKDKPLFNQAMCWCTDTPDGHLLICEHPEWKNFYLATGDSGHSFKLLPIIGKYVVELLEGTLADELAHKWRWRPGSGDALKSRREAPAKDLADMPGWNHD

>P7

MAPSRKNTKVIVVGGGGTIGSSTALHLVRSGYTPSNITVLDAYPIPSSQSAGNDLNKIMDADADPAADAARQMWKNDPLFKPFFHNTGRLDCAHGEKGIAELKKRYQNLVDAGQAGNVEWLDSEDEILKRMPQLTRDQIKGWKAIFSKDGGWLAAAKAIKAIGEYLRDQGVRFGFGGAGSFKQPLLENGVCIGVETVDGTRYYADKVVLAAGAWSPTLVDLQGQCVSKAWVYGHIQLTPEEAARYKNCPVVYNGDVGFFFEPNEDGIIKVCDEFPGFTNFKMHQPFGAPHPKRISVPRSHAKHPTDTIPDEAEERIRRAIRTFMPQFKDKPLFNQAMCWCTDTPDGHLLICEHPEWKNFYLATGDSGHSFKLLPIIGKYVVELLEGTLADELAHKWRWRPGSGDALKSRREAPAKDLSDMPGWNHD

>P8

MAPSRKNTRVIVVGGGGTIGSSTALHLVRSGYTPSNITVLDAYPIPSSQSAGNDLNKIMDADADPAADAARDMWKNDPLFKPFFHNTGILDCAHGEKGIAELRKRYQNLVDAGQGGNVEWLDSEDEILKRMPQLTRDQIKGWKAIFSKDGGWLAAAKAIKAIGEYLRDMGVRFGFGGAGSFKQPLLENGVCIGVETVDGTRYYADKVVLAAGAWSPTLVDLKGQCVSKAWTYGHIQLTPEEAARYKNCPVVYNGDVGFFFEPNEDGIIKVCDEFPGFTNFTMHQPFGAPHPKRISVPRSHAKHPTDTIPDEARQRIRKAIRTFMPQFADKPLFNQAMCWCTDTPDGHLLICEHPEWKNFYLATGDSGHSFKLLPVIGKYVVELLEGTLADELAHKWRWRPGSGDALKSRREAPAKDLSDMPGWNHD

>P9

MAPSRKDTRVIVVGGGGTIGSSTALHLVRSGYTPSNITVLDAYPIPSSQSAGNDLNKIMDADADPAADAARDMWKNDPLFKPFFHNTGILDCAHGPEGIAELRKRYQNLKDAGQGGNIEWLDSEDEILKRMPQLTRDQIKGWKAIFNKDGGWLAAAKAIKAIGEELRRQGVKFGFGGSGSFKQPILENGDCIGVETVDGTRYYADKVVLAAGAWSPTLVDLKGQCVAKAWTYGHIQLTPEEAARYKNMPVVYNGDVGFFFEPNEDGIIKICDEFPGYTNYTMHQPFGAPHPKRISVPRSHAKHPTDTIPDEAEKRIRKAIRTFMPQFADKPLFNQAMCWCTDTPDGHLLICEHPKWKNFYLATGDSGHSFKLLPVIGKYVVELLEGTLPEELAHKWRWRPGSGDALKSRRAAPAKDLSDMPGWNHD

>S01

MAPSRANTKVIVVGGGGTIGSSTALHLVRSGYTPSNVTVLDAYPIPSSQSAGNDLNKIMDADADPAADAARQMWNEDELFKKFFHNTGRLDCAHGEKDIADLKKRYQNLVDWGLSATVEWLDSEDEILKRMPQLTRDQIKGWKAIFSKDGGWLAAAKAIKAIGEYLRDQGVRFGFYGAGSFKQPLLAEGVCIGVETVDGTRYYADKVVLAAGAWSPTLVELQEQCVSKAWVYGHIQLTPEEAARYKNSPVVYNGDVGFFFEPNEHGIIKVCDEFPGFTRFKMHQPFGAKAPKRISVPRSHAKHPTDTIPDASIVRIRRAIATFMPQFKNKPLFNQAMCWCTDTADGHLLICEHPEWKNFYLATGDSGDSFKLLPIIGKYVVELLEGTLADELAHKWRWRPGSGDALKSRREAPAKDLADMPGWNHD

>S02

MAPSRANTKVIVVGGGGTIGSSTALHLVRSGYTPSNVTVLDAYPIPSSQSAGNDLNKIMDADADPAADAARQMWNEDELFKKFFHNTGRLDCAHGEKDIADLKKRYQNLVDGGLDATVEWLDSEDEILKRMPQLTRDQIKGWKAIFSKDGGWLAAAKAIKAIGEYLRDQGVRFGFYGAGSFKQPLLAEGVCIGVETVDGTRYYADKVVLAAGAWSPTLVELQEQCVSKAWVYGHIQLTPEEAARYKNSPVVYNGDVGFFFEPNEHGIIKVCDEFPGFTRFKMHQPFGAKAPKRISVPRSHAKHPTDTIPDASIVRIRRAIATFMPQFKNKPLFNQAMCWCTDTADGHLLICEHPEWKNFYLATGDSGDSFKLLPIIGKYVVELLEGTLADELAHKWRWRPGSGDALKSRREAPAKDLADMPGWNHD

>S03

MAPSRANTKVIVVGGGGTIGSSTALHLVRSGYTPSNVTVLDAYPIPSSQSAGNDLNKIMDADADPAADAARQMWNEDELFKKFFHNTGRLDCAHGEKDIADLKKRYQNLVDWGLGATVEWLDSEDEILKRMPQLTRDQIKGWKAIFSKDGGWLAAAKAIKAIGEYLRDQGVRFGFYGAGSFKQPLLAEGVCIGVETVDGTRYYADKVVLAAGAWSPTLVELQEQCVSKAWVYGHIQLTPEEAARYKNSPVVYNGDVGFFFEPNEHGIIKVCDEFPGFTRFKMHQPFGAKAPKRISVPRSHAKHPTDTIPDASIVRIRRAIATFMPQFKNKPLFNQAMCWCTDTADGHLLICEHPEWKNFYLATGDSGDSFKLLPIIGKYVVELLEGTLADELAHKWRWRPGSGDALKSRREAPAKDLADMPGWNHD

>S04

MAPSRANTKVIVVGGGGTIGSSTALHLVRSGYTPSNVTVLDAYPIPSSQSAGNDLNKIMDADADPAADAARQMWNEDELFKKFFHNTGRLDCAHGEKDIADLKKRYQNLVDWGLAATVEWLDSEDEILKRMPQLTRDQIKGWKAIFSKDGGWLAAAKAIKAIGEYLRDQGVRFGFYGAGSFKQPLLAEGVCIGVETVDGTRYYADKVVLAAGAWSPTLVELQEQCVSKAWVYGHIQLTPEEAARYKNSPVVYNGDVGFFFEPNEHGIIKVCDEFPGFTRFKMHQPFGAKAPKRISVPRSHAKHPTDTIPDASIVRIRRAIATFMPQFKNKPLFNQAMCWCTDTADGHLLICEHPEWKNFYLATGDSGDSFKLLPIIGKYVVELLEGTLADELAHKWRWRPGSGDALKSRREAPAKDLADMPGWNHD

>S05

MAPSRANTKVIVVGGGGTIGSSTALHLVRSGYTPSNVTVLDAYPIPSSQSAGNDLNKIMDADADPAADAARQMWNEDELFKKFFHNTGRLDCAHGEKDIADLKKRYQNLVDWGGDATVEWLDSEDEILKRMPQLTRDQIKGWKAIFSKDGGWLAAAKAIKAIGEYLRDQGVRFGFYGAGSFKQPLLAEGVCIGVETVDGTRYYADKVVLAAGAWSPTLVELQEQCVSKAWVYGHIQLTPEEAARYKNSPVVYNGDVGFFFEPNEHGIIKVCDEFPGFTRFKMHQPFGAKAPKRISVPRSHAKHPTDTIPDASIVRIRRAIATFMPQFKNKPLFNQAMCWCTDTADGHLLICEHPEWKNFYLATGDSGDSFKLLPIIGKYVVELLEGTLADELAHKWRWRPGSGDALKSRREAPAKDLADMPGWNHD

>S06

MAPSRANTKVIVVGGGGTIGSSTALHLVRSGYTPSNVTVLDAYPIPSSQSAGNDLNKIMDADADPAADAARQMWNEDELFKKFFHNTGRLDCAHGEKDIADLKKRYQNLVDWGLDETVEWLDSEDEILKRMPQLTRDQIKGWKAIFSKDGGWLAAAKAIKAIGEYLRDQGVRFGFYGAGSFKQPLLAEGVCIGVETVDGTRYYADKVVLAAGAWSPTLVELQEQCVSKAWVYGHIQLTPEEAARYKNSPVVYNGDVGFFFEPNEHGIIKVCDEFPGFTRFKMHQPFGAKAPKRISVPRSHAKHPTDTIPDASIVRIRRAIATFMPQFKNKPLFNQAMCWCTDTADGHLLICEHPEWKNFYLATGDSGDSFKLLPIIGKYVVELLEGTLADELAHKWRWRPGSGDALKSRREAPAKDLADMPGWNHD

>S07

MAPSRANTKVIVVGGGGTIGSSTALHLVRSGYTPSNVTVLDAYPIPSSQSAGNDLNKIMDADADPAADAARQMWNEDELFKKFFHNTGRLDCAHGEKDIADLKKRYQNLVDTGLDATVEWLDSEDEILKRMPQLTRDQIKGWKAIFSKDGGWLAAAKAIKAIGEYLRDQGVRFGFYGAGSFKQPLLAEGVCIGVETVDGTRYYADKVVLAAGAWSPTLVELQEQCVSKAWVYGHIQLTPEEAARYKNSPVVYNGDVGFFFEPNEHGIIKVCDEFPGFTRFKMHQPFGAKAPKRISVPRSHAKHPTDTIPDASIVRIRRAIATFMPQFKNKPLFNQAMCWCTDTADGHLLICEHPEWKNFYLATGDSGDSFKLLPIIGKYVVELLEGTLADELAHKWRWRPGSGDALKSRREAPAKDLADMPGWNHD

>S08

MAPSRANTKVIVVGGGGTIGSSTALHLVRSGYTPSNVTVLDAYPIPSSQSAGNDLNKIMDADADPAADAARQMWNEDELFKKFFHNTGRLDCAHGEKDIADLKKRYQNLVDWGLDKTVEWLDSEDEILKRMPQLTRDQIKGWKAIFSKDGGWLAAAKAIKAIGEYLRDQGVRFGFYGAGSFKQPLLAEGVCIGVETVDGTRYYADKVVLAAGAWSPTLVELQEQCVSKAWVYGHIQLTPEEAARYKNSPVVYNGDVGFFFEPNEHGIIKVCDEFPGFTRFKMHQPFGAKAPKRISVPRSHAKHPTDTIPDASIVRIRRAIATFMPQFKNKPLFNQAMCWCTDTADGHLLICEHPEWKNFYLATGDSGDSFKLLPIIGKYVVELLEGTLADELAHKWRWRPGSGDALKSRREAPAKDLADMPGWNHD

>S09

MAPSRANTKVIVVGGGGTIGSSTALHLVRSGYTPSNVTVLDAYPIPSSQSAGNDLNKIMDADADPAADAARQMWNEDELFKKFFHNTGRLDCAHGEKDIADLKKRYQNLVDSGLDATVEWLDSEDEILKRMPQLTRDQIKGWKAIFSKDGGWLAAAKAIKAIGEYLRDQGVRFGFYGAGSFKQPLLAEGVCIGVETVDGTRYYADKVVLAAGAWSPTLVELQEQCVSKAWVYGHIQLTPEEAARYKNSPVVYNGDVGFFFEPNEHGIIKVCDEFPGFTRFKMHQPFGAKAPKRISVPRSHAKHPTDTIPDASIVRIRRAIATFMPQFKNKPLFNQAMCWCTDTADGHLLICEHPEWKNFYLATGDSGDSFKLLPIIGKYVVELLEGTLADELAHKWRWRPGSGDALKSRREAPAKDLADMPGWNHD

>S10

MAPSRANTKVIVVGGGGTIGSSTALHLVRSGYTPSNVTVLDAYPIPSSQSAGNDLNKIMDADADPAADAARQMWNEDELFKKFFHNTGRLDCAHGEKDIADLKKRYQNLVDDGLDATVEWLDSEDEILKRMPQLTRDQIKGWKAIFSKDGGWLAAAKAIKAIGEYLRDQGVRFGFYGAGSFKQPLLAEGVCIGVETVDGTRYYADKVVLAAGAWSPTLVELQEQCVSKAWVYGHIQLTPEEAARYKNSPVVYNGDVGFFFEPNEHGIIKVCDEFPGFTRFKMHQPFGAKAPKRISVPRSHAKHPTDTIPDASIVRIRRAIATFMPQFKNKPLFNQAMCWCTDTADGHLLICEHPEWKNFYLATGDSGDSFKLLPIIGKYVVELLEGTLADELAHKWRWRPGSGDALKSRREAPAKDLADMPGWNHD

>S11

MAPSRANTKVIVVGGGGTIGSSTALHLVRSGYTPSNVTVLDAYPIPSSQSAGNDLNKIMDADADPAADAARQMWNEDELFKKFFHNTGRLDCAHGEKDIADLKKRYQNLVDNGLDATVEWLDSEDEILKRMPQLTRDQIKGWKAIFSKDGGWLAAAKAIKAIGEYLRDQGVRFGFYGAGSFKQPLLAEGVCIGVETVDGTRYYADKVVLAAGAWSPTLVELQEQCVSKAWVYGHIQLTPEEAARYKNSPVVYNGDVGFFFEPNEHGIIKVCDEFPGFTRFKMHQPFGAKAPKRISVPRSHAKHPTDTIPDASIVRIRRAIATFMPQFKNKPLFNQAMCWCTDTADGHLLICEHPEWKNFYLATGDSGDSFKLLPIIGKYVVELLEGTLADELAHKWRWRPGSGDALKSRREAPAKDLADMPGWNHD

>S12

MAPSRANTKVIVVGGGGTIGSSTALHLVRSGYTPSNVTVLDAYPIPSSQSAGNDLNKIMDADADPAADAARQMWNEDELFKKFFHNTGRLDCAHGEKDIADLKKRYQNLVDWGLDGTVEWLDSEDEILKRMPQLTRDQIKGWKAIFSKDGGWLAAAKAIKAIGEYLRDQGVRFGFYGAGSFKQPLLAEGVCIGVETVDGTRYYADKVVLAAGAWSPTLVELQEQCVSKAWVYGHIQLTPEEAARYKNSPVVYNGDVGFFFEPNEHGIIKVCDEFPGFTRFKMHQPFGAKAPKRISVPRSHAKHPTDTIPDASIVRIRRAIATFMPQFKNKPLFNQAMCWCTDTADGHLLICEHPEWKNFYLATGDSGDSFKLLPIIGKYVVELLEGTLADELAHKWRWRPGSGDALKSRREAPAKDLADMPGWNHD

>S13

MAPSRANTKVIVVGGGGTIGSSTALHLVRSGYTPSNVTVLDAYPIPSSQSAGNDLNKIMDADADPAADAARQMWNEDELFKKFFHNTGRLDCAHGEKDIADLKKRYQNLRDWGLDATVEWLDSEDEILKRMPQLTRDQIKGWKAIFSKDGGWLAAAKAIKAIGEYLRDQGVRFGFYGAGSFKQPLLAEGVCIGVETVDGTRYYADKVVLAAGAWSPTLVELQEQCVSKAWVYGHIQLTPEEAARYKNSPVVYNGDVGFFFEPNEHGIIKVCDEFPGFTRFKMHQPFGAKAPKRISVPRSHAKHPTDTIPDASIVRIRRAIATFMPQFKNKPLFNQAMCWCTDTADGHLLICEHPEWKNFYLATGDSGDSFKLLPIIGKYVVELLEGTLADELAHKWRWRPGSGDALKSRREAPAKDLADMPGWNHD

>S14

MAPSRANTKVIVVGGGGTIGSSTALHLVRSGYTPSNVTVLDAYPIPSSQSAGNDLNKIMDADADPAADAARQMWNEDELFKKFFHNTGRLDCAHGEKDIADLKKRYQNLVDWGLDTTVEWLDSEDEILKRMPQLTRDQIKGWKAIFSKDGGWLAAAKAIKAIGEYLRDQGVRFGFYGAGSFKQPLLAEGVCIGVETVDGTRYYADKVVLAAGAWSPTLVELQEQCVSKAWVYGHIQLTPEEAARYKNSPVVYNGDVGFFFEPNEHGIIKVCDEFPGFTRFKMHQPFGAKAPKRISVPRSHAKHPTDTIPDASIVRIRRAIATFMPQFKNKPLFNQAMCWCTDTADGHLLICEHPEWKNFYLATGDSGDSFKLLPIIGKYVVELLEGTLADELAHKWRWRPGSGDALKSRREAPAKDLADMPGWNHD

>S15

MAPSRANTKVIVVGGGGTIGSSTALHLVRSGYTPSNVTVLDAYPIPSSQSAGNDLNKIMDADADPAADAARQMWNEDELFKKFFHNTGRLDCAHGEKDIADLKKRYQNLVDWGLDATVEWLDSEDEILKRMPQLTRDQIKGWKAIFSKDGGWLAAAKAIKAIGEYLRDQGVRFGFYGAGSFKQPLLAEGVCIGVETVDGTRYYADKVVLAAGAWSYTLVELQEQCVSKAWVYGHIQLTPEEAARYKNSPVVYNGDVGFFFEPNEHGIIKVCDEFPGFTRFKMHQPFGAKAPKRISVPRSHAKHPTDTIPDASIVRIRRAIATFMPQFKNKPLFNQAMCWCTDTADGHLLICEHPEWKNFYLATGDSGDSFKLLPIIGKYVVELLEGTLADELAHKWRWRPGSGDALKSRREAPAKDLADMPGWNHD

>S16

MAPSRANTKVIVVGGGGTIGSSTALHLVRSGYTPSNVTVLDAYPIPSSQSAGNDLNKIMDADADPAADAARQMWNEDELFKKFFHNTGRLDCAHGEKDIADLKKRYQNLHDWGLDATVEWLDSEDEILKRMPQLTRDQIKGWKAIFSKDGGWLAAAKAIKAIGEYLRDQGVRFGFYGAGSFKQPLLAEGVCIGVETVDGTRYYADKVVLAAGAWSPTLVELQEQCVSKAWVYGHIQLTPEEAARYKNSPVVYNGDVGFFFEPNEHGIIKVCDEFPGFTRFKMHQPFGAKAPKRISVPRSHAKHPTDTIPDASIVRIRRAIATFMPQFKNKPLFNQAMCWCTDTADGHLLICEHPEWKNFYLATGDSGDSFKLLPIIGKYVVELLEGTLADELAHKWRWRPGSGDALKSRREAPAKDLADMPGWNHD

>S17

MAPSRANTKVIVVGGGGTIGSSTALHLVRSGYTPSNVTVLDAYPIPSSQSAGNDLNKIMDADADPAADAARQMWNEDELFKKFFHNTGRLDCAHGEKDIADLKKRYQNLVDWGLDATVEWLDSEDEILKRMPQLTRDQIKGWKAIFSKDGGWLAAAKAIKAIGEYLRDQGVRFGFYGAGSFKQPLLAEGVCIGVETVDGTRYYADKVVLAAGAWSATLVELQEQCVSKAWVYGHIQLTPEEAARYKNSPVVYNGDVGFFFEPNEHGIIKVCDEFPGFTRFKMHQPFGAKAPKRISVPRSHAKHPTDTIPDASIVRIRRAIATFMPQFKNKPLFNQAMCWCTDTADGHLLICEHPEWKNFYLATGDSGDSFKLLPIIGKYVVELLEGTLADELAHKWRWRPGSGDALKSRREAPAKDLADMPGWNHD

>S18

MAPSRANTKVIVVGGGGTIGSSTALHLVRSGYTPSNVTVLDAYPIPSSQSAGNDLNKIMDADADPAADAARQMWNEDELFKKFFHNTGRLDCAHGEKDIADLKKRYQNLKDWGLDATVEWLDSEDEILKRMPQLTRDQIKGWKAIFSKDGGWLAAAKAIKAIGEYLRDQGVRFGFYGAGSFKQPLLAEGVCIGVETVDGTRYYADKVVLAAGAWSPTLVELQEQCVSKAWVYGHIQLTPEEAARYKNSPVVYNGDVGFFFEPNEHGIIKVCDEFPGFTRFKMHQPFGAKAPKRISVPRSHAKHPTDTIPDASIVRIRRAIATFMPQFKNKPLFNQAMCWCTDTADGHLLICEHPEWKNFYLATGDSGDSFKLLPIIGKYVVELLEGTLADELAHKWRWRPGSGDALKSRREAPAKDLADMPGWNHD

>S19

MAPSRANTKVIVVGGGGTIGSSTALHLVRSGYTPSNVTVLDAYPIPSSQSAGNDLNKIMDADADPAADAARQMWNEDELFKKFFHNTGRLDCAHGEKDIADLKKRYQNLVDWGLDATVEWLDSEDEILKRMPQLTRDQIKGWKAIFSKDGGWLAAAKAIKAIGEYLRDQGVRFGFYGAGSFKQPLLAEGVCIGVETVDGTRYYADKVVLAAGAWSSTLVELQEQCVSKAWVYGHIQLTPEEAARYKNSPVVYNGDVGFFFEPNEHGIIKVCDEFPGFTRFKMHQPFGAKAPKRISVPRSHAKHPTDTIPDASIVRIRRAIATFMPQFKNKPLFNQAMCWCTDTADGHLLICEHPEWKNFYLATGDSGDSFKLLPIIGKYVVELLEGTLADELAHKWRWRPGSGDALKSRREAPAKDLADMPGWNHD

>S20

MAPSRANTKVIVVGGGGTIGSSTALHLVRSGYTPSNVTVLDAYPIPSSQSAGNDLNKIMDADADPAADAARQMWNEDELFKKFFHNTGRLDCAHGEKDIADLKKRYQNLGDWGLDATVEWLDSEDEILKRMPQLTRDQIKGWKAIFSKDGGWLAAAKAIKAIGEYLRDQGVRFGFYGAGSFKQPLLAEGVCIGVETVDGTRYYADKVVLAAGAWSPTLVELQEQCVSKAWVYGHIQLTPEEAARYKNSPVVYNGDVGFFFEPNEHGIIKVCDEFPGFTRFKMHQPFGAKAPKRISVPRSHAKHPTDTIPDASIVRIRRAIATFMPQFKNKPLFNQAMCWCTDTADGHLLICEHPEWKNFYLATGDSGDSFKLLPIIGKYVVELLEGTLADELAHKWRWRPGSGDALKSRREAPAKDLADMPGWNHD

>X01

MAPSRANTKVIVVGGGGTIGSSTALHLVRSGYTPSNVTVLDAYPIPSSCSAGNDLNKIMDADADPAADAARQMWNEDELFKKFFHNTGRLDCAHGEKDIADLKKRYQNLVDWGLDATVEWLDSEDEILKRMPQLTRDQIKGWKAIFSKDGGWLAAAKAIKAIGEYLRDQGVRFGFYGAGSFKQPLLAEGVCIGVETVDGTRYYADKVVLAAGAWSPTLVELQEQCVSKAWVYGHIQLTPEEAARYKNSPVVYNGDVGFFFEPNEHGIIKVCDEFPGFTRFKMHQPFGAKAPKRISVPRSHCKHPTDTIPDASIVRIRRAIATFMPQFKNKPLFNQAMCWCTDTADGHLLICEHPEWKNFYLATGDSGDSFKLLPIIGKYVVELLEGTLADELAHKWRWRPGSGDALKSRREAPAKDLADMPGWNHD

>X02

MAPSRANTKVIVVGGGGTIGSSTALHLVRSGYTPSNVTVLDAYPIPSCQSAGNDLNKIMDADADPAADAARQMWNEDELFKKFFHNTGRLDCAHGEKDIADLKKRYQNLVDWGLDATVEWLDSEDEILKRMPQLTRDQIKGWKAIFSKDGGWLAAAKAIKAIGEYLRDQGVRFGFYGAGSFKQPLLAEGVCIGVETVDGTRYYADKVVLAAGAWSPTLVELQEQCVSKAWVYGHIQLTPEEAARYKNSPVVYNGDVGFFFEPNEHGIIKVCDEFPGFTRFKMHQPFGAKAPKRISVPRSHCKHPTDTIPDASIVRIRRAIATFMPQFKNKPLFNQAMCWCTDTADGHLLICEHPEWKNFYLATGDSGDSFKLLPIIGKYVVELLEGTLADELAHKWRWRPGSGDALKSRREAPAKDLADMPGWNHD

>X03

MAPSRANTKVIVVGGGGTIGSSTALHLVRSGYTPSNVTVLDAYPIPSSQSAGNDLNKIMDADADPAADAARQMWNEDELFKKFFHNTGRLDCCHGEKCIADLKKRYQNLVDWGLDATVEWLDSEDEILKRMPQLTRDQIKGWKAIFSKDGGWLAAAKAIKAIGEYLRDQGVRFGFYGAGSFKQPLLAEGVCIGVETVDGTRYYADKVVLAAGAWSPTLVELQEQCVSKAWVYGHIQLTPEEAARYKNSPVVYNGDVGFFFEPNEHGIIKVCDEFPGFTRFKMHQPFGAKAPKRISVPRSHAKHPTDTIPDASIVRIRRAIATFMPQFKNKPLFNQAMCWCTDTADGHLLICEHPEWKNFYLATGDSGDSFKLLPIIGKYVVELLEGTLADELAHKWRWRPGSGDALKSRREAPAKDLADMPGWNHD

>X04

MAPSRANTKVIVVGGGGTIGSSTALHLVRSGYTPSNVTVLDAYPIPSSQSAGNDLNKIMDADADPAADAARQMWNEDELFKKFFHNTGRLDCCHGEKDIADCKKRYQNLVDWGLDATVEWLDSEDEILKRMPQLTRDQIKGWKAIFSKDGGWLAAAKAIKAIGEYLRDQGVRFGFYGAGSFKQPLLAEGVCIGVETVDGTRYYADKVVLAAGAWSPTLVELQEQCVSKAWVYGHIQLTPEEAARYKNSPVVYNGDVGFFFEPNEHGIIKVCDEFPGFTRFKMHQPFGAKAPKRISVPRSHAKHPTDTIPDASIVRIRRAIATFMPQFKNKPLFNQAMCWCTDTADGHLLICEHPEWKNFYLATGDSGDSFKLLPIIGKYVVELLEGTLADELAHKWRWRPGSGDALKSRREAPAKDLADMPGWNHD

>X05

MAPSRANTKVIVVGGGGTIGSSTALHLVRSGYTPSNVTVLDAYPIPSSQSAGNDLNKIMDADADPAADAARQMWNEDELFKKFFHNTGRLDCAHGEKDIADLKKRYQNLVDWGLDATVEWLDSEDEILKRMPQLTRDQIKGWKAIFSKDGGWLAAAKAIKAIGEYLRDQGVRFGFYGAGSFKQPLLAEGVCIGVETVDGTRYYADKVVLAAGAWSPTLVELQEQCVSKAWVYGHIQCTPECAARYKNSPVVYNGDVGFFFEPNEHGIIKVCDEFPGFTRFKMHQPFGAKAPKRISVPRSHAKHPTDTIPDASIVRIRRAIATFMPQFKNKPLFNQAMCWCTDTADGHLLICEHPEWKNFYLATGDSGDSFKLLPIIGKYVVELLEGTLADELAHKWRWRPGSGDALKSRREAPAKDLADMPGWNHD

>X06

MAPSRANTKVIVVGGGGTIGSSTALHLVRSGYTPSNVTVLDAYPIPSSQSAGNDLNKIMDADADPAADAARQMWNEDELFKKFFHNTGRLDCAHGEKDIADLKKRYQNLVDWGLDATVEWLDSEDEILKRMPQLTRDQIKGWKAIFSKDGGWLAAAKAIKAIGEYLRDQGVRFGFYGAGSFKQPLLAEGVCIGVETVDGTRYYADKVVLAAGAWSPTLVELQEQCVSKAWVYGHIQCTPEECARYKNSPVVYNGDVGFFFEPNEHGIIKVCDEFPGFTRFKMHQPFGAKAPKRISVPRSHAKHPTDTIPDASIVRIRRAIATFMPQFKNKPLFNQAMCWCTDTADGHLLICEHPEWKNFYLATGDSGDSFKLLPIIGKYVVELLEGTLADELAHKWRWRPGSGDALKSRREAPAKDLADMPGWNHD

>X07

MAPSRANTKVIVVGGGGTIGSSTALHLVRSGYTPSNVTVLDAYPIPSSQSAGNDLNKIMDADADPAADAARQMWNEDELFKKFFHNTGRLDCAHGEKDIADLKKRYQNLVDWGLDATVEWLDSEDEILKRMPQLTRDQIKGWKAIFSKDGGWLAAAKAIKAIGEYLRDQGVRFGFYGAGSFKQPLLAEGVCIGVETVDGTRYYADKVVLAAGAWSPTLVELQEQCVSKAWVYGHIQLTPEECARYKNSPVVYNGDVGFFFEPNEHCIIKVCDEFPGFTRFKMHQPFGAKAPKRISVPRSHAKHPTDTIPDASIVRIRRAIATFMPQFKNKPLFNQAMCWCTDTADGHLLICEHPEWKNFYLATGDSGDSFKLLPIIGKYVVELLEGTLADELAHKWRWRPGSGDALKSRREAPAKDLADMPGWNHD

>X08

MAPSRANTKVIVVGGGGTIGSSTALHLVRSGYTPSNVTVLDAYPIPSSQSAGNDLNKIMDADADPAADAARQMWNEDELFKKFFHNTGRLDCAHGEKDIADLKKRYQNLVDWGLDATVEWLDSEDEILKRMPQLTRDQIKGWKAIFSKDGGWLAAAKAIKAIGEYLRDQGVRFGFYGAGSFKQPLLAEGVCIGVETVDGTRYYADKVVLAAGAWSPTLVELQEQCVSKAWVYGHIQLTPEEAARYKNSPVVYNGDVGFFFEPNEHGIIKVCDEFPGFTRFKMHQPFGAKAPKRISVPRCHACHPTDTIPDASIVRIRRAIATFMPQFKNKPLFNQAMCWCTDTADGHLLICEHPEWKNFYLATGDSGDSFKLLPIIGKYVVELLEGTLADELAHKWRWRPGSGDALKSRREAPAKDLADMPGWNHD

**References**

1. Bond, PS. JTSA. (2017). at http://paulsbond.co.uk/jtsa
